# *Clostridium butyricum* Prazmowski can degrade and utilize resistant starch via a set of synergistically acting enzymes

**DOI:** 10.1128/msphere.00566-23

**Published:** 2023-12-22

**Authors:** Tara L. Pickens, Darrell W. Cockburn

**Affiliations:** 1 Department of Food Science, The Pennsylvania State University, State College, Pennsylvania, USA; 2 The One Health Microbiome Center, Huck Institute of the Life Sciences, The Pennsylvania State University, State College, Pennsylvania, USA; University of Michigan-Ann Arbor, Ann Arbor, Michigan, USA

**Keywords:** resistant starch, probiotic, synbiotic, butyrate, enzymes

## Abstract

Resistant starch is a prebiotic fiber that is best known for its ability to increase butyrate production by the gut microbiota. This butyrate then plays an important role in modulating the immune system and inflammation. However, the ability to use this resistant starch appears to be a rare trait within the gut microbiota, with only a few species such as *Ruminococcus bromii* and *Bifidobacterium adolescentis* having been demonstrated to possess this ability. Furthermore, these bacteria do not directly produce butyrate themselves, rather they rely on cross-feeding interactions with other gut bacteria for its production. Here, we demonstrate that the often-used probiotic organism *Clostridium butyricum* also possesses the ability to utilize resistant starch from a number of sources, with direct production of butyrate. We further explore the enzymes responsible for this trait, demonstrating that they exhibit significant synergy, though with different enzymes exhibiting more or less importance depending on the source of the resistant starch. Thus, the co-administration of *Clostridium butyricum* may have the ability to improve the beneficial effects of resistant starch.

**IMPORTANCE:**

*Clostridium butyricum* is seeing increased use as a probiotic, due to potential health benefits tied to its ability to produce butyrate. Here, we demonstrate that this organism can use a variety of resistant starch sources and characterize the enzymes it uses to accomplish this. Given the relative rarity of resistant starch utilizing ability within the gut and the health benefits tied to resistant starch, the combined use of this organism with resistant starch in synbiotic formulations may prove beneficial.

## INTRODUCTION

There is now a wealth of evidence to conclude that the composition and metabolic output of the gut microbiota rely heavily on the host’s diet and that its members play integral roles in mediating physical and mental health (1–5). An important mechanism in supporting these attributes in most individuals is a diet rich in dietary fiber, which consists of complex carbohydrates that are not digestible by human enzymes, and thus these food components reach the lower gastrointestinal tract unscathed, where they serve as a critical food source for gut microorganisms (6). The metabolites produced from this fermentation play myriad roles in the body, from regulating metabolism and the immune system to impacting mood and supporting brain health, to improving pathogen colonization resistance (4, 7–10). Short-chain fatty acids, particularly butyrate, have been consistently shown to exert various health-promoting effects (11, 12), acting as inhibitors of histone deacetylases and/or activators of G-protein-coupled receptors (11). Within the broad category of dietary fiber, a particular type called resistant starch (RS), which for many chemical and physical reasons evades human digestive enzymes and transits to the colon intact, is a key butyrogenic substrate (13, 14). Despite evidence to support this, efforts to employ RS to promote butyrate formation *in vivo* have yielded mixed results, with strong interindividual variation in response to dietary intervention (15, 16). This is tied to differences in the composition of an individual’s microbiota, which could vary in its capacity to degrade RS and/or utilize those degradation products to generate butyrate. Furthermore, *in vitro* studies have shown variation in the ability of RS-degrading microorganisms to tackle several types of RS structures (15). One way to overcome these differences and reap the health benefits linked to butyrate production in the lower gastrointestinal tract would be to employ a complementary combination of RS degrader and RS to promote butyrate formation; however, there is still much unknown about the RS-degrading landscape of the human gut.

The production of RS-degrading enzymes is a rare trait within the human gut microbiota, with only *Bifidobacterium adolescentis* and a few closely related *Bifidobacterium* species (17) as well as *Ruminococcus bromii* (18) and the recently described *Ruminococcus* FMB-CY1 (19) are known to possess this trait. This represents much less than 1% of the more than 3,500 known species found within the human gut (20), suggesting that this limited set of species are important keystone members of the gut microbiota. *B. adolescentis* and *R. bromii* share the commonality of expressing several enzymes that are localized to the cell surface and consist of multiple α-amylase and/or pullulanase enzymatic domains, as well as carbohydrate-binding modules (CBMs) that enable the attachment of the enzyme and organism to the starch granule surface while the enzymes catalyze the hydrolysis of the substrate. The production of butyrate from RS degradation by these two microorganisms occurs indirectly, through the cross-feeding of RS degradation products, liberated oligosaccharides, and short-chain fatty acids like acetate and lactate to butyrate-producing gut commensals (21). It is highly likely that other modes of RS degradation exist in this ecosystem but have not yet been elucidated. First, it is possible that gut microorganisms expressing extracellular enzyme systems unable to tackle RS on their own can work synergistically with other gut microorganisms possessing a complementary enzyme system to degrade these recalcitrant substrates. Another possibility is that there are other unstudied gut microorganisms that have evolved similar specialized enzymatic machinery that can attach to and disassemble RS granules. For instance, it has been shown that dietary supplementation with RS from potatoes results in the increase of *R. bromii* or *Clostridium chartatabidum*, along with the concomitant increase in butyrate concentration in the feces (16). Additionally, the relative abundance of *Parabacteroides distasonis* has been shown to increase, along with the short-chain fatty acid propionate, in response to a dietary intervention of cross-linked tapioca starch, a chemically modified (Type 4) resistant starch (15). However, more work needs to be done to determine if these organisms can efficiently degrade RS. Aside from these studies, there could be other RS-degrading organisms that are present at low abundance, with strong responses to other dietary factors, or are too different from known bacterial species to be identified or have a highly similar 16S rRNA sequence to one or more other bacteria in the microbiota, causing them to be missed in typical intervention studies. Furthermore, there are many different structural variations of RS, and it could be possible that the correct combinations of RS and individual microbiota have not been clinically tested to identify all RS-degrading bacteria in the human gut.

Since the strategy of increasing butyrate *in vivo* through dietary supplementation with RS seems to be highly dependent on the individual’s microbiota, it has not found wide utility as a therapeutic intervention as it is currently unknown what microbes are the key drivers of increased butyrate production from each RS source. It could be possible to increase butyrate production *in vivo* by utilizing a microorganism with both the capacity to degrade RS and to produce butyrate with a synbiotic combination of RS sources. One such organism is the commonly employed probiotic, *Clostridium butyricum*, which is a strictly anaerobic, spore-forming bacterium ubiquitous in nature, where it is found in a variety of ecosystems, including the mammalian gut. It was first isolated from pig intestines by Prazmowski in 1880 (22), and subsequently it was found to produce an extracellular amylase during growth on soluble potato starch (23). Another study identified a raw potato starch binding α-amylase of MW 80,000 kDa, which was detected in the supernatant of the spent growth media when *C. butyricum* was grown on various types of raw starch granules, which cleaved soluble starch to maltose and maltotriose (24). In Asia, *C. butyricum* MIYAIRI 588 (CBM 588) has been administered as a probiotic since the 1960s due to its ability to produce organic acids, particularly butyrate, and its role in colonization resistance has been more recently elucidated (25, 26). Intriguingly, studies have shown the potential for this microorganism to protect against certain diseases like hypertension and pancreatic disease, which, through various mechanisms, is at least partially due to its production of butyrate (27, 28). Furthermore, this microorganism has been shown to persist in the gastrointestinal tracts of rats for up to 6 days post-ingestion, indicating the potential for its ability to exert positive effects with regular administration (29).

However, despite its frequent use as a probiotic, this RS-degrading capability of *C. butyricum* is rarely mentioned and its enzymatic machinery for doing this remains largely unexplored. We hypothesized that *C. butyricum* is capable of growth on a variety of RS substrates and contains a suite of enzymes for RS digestion akin to what is seen in the other gut RS degraders *R. bromii* and *B. adolescentis*. To test this, we investigated the growth and organic acid production of *C. butyricum* on several starch sources. We then examined the genome for enzymes likely to contribute to RS digestion, produced them recombinantly in *Escherichia coli*, and characterized their activity both alone and in combination. Together, these results demonstrate that *C. butyricum* is indeed an efficient utilizer of multiple RS sources, which has important implications for its use as a probiotic in both humans and animals (30).

## RESULTS

### Characterization of the bacteria

#### Monitoring growth via organic acid production

**Fig 1:**
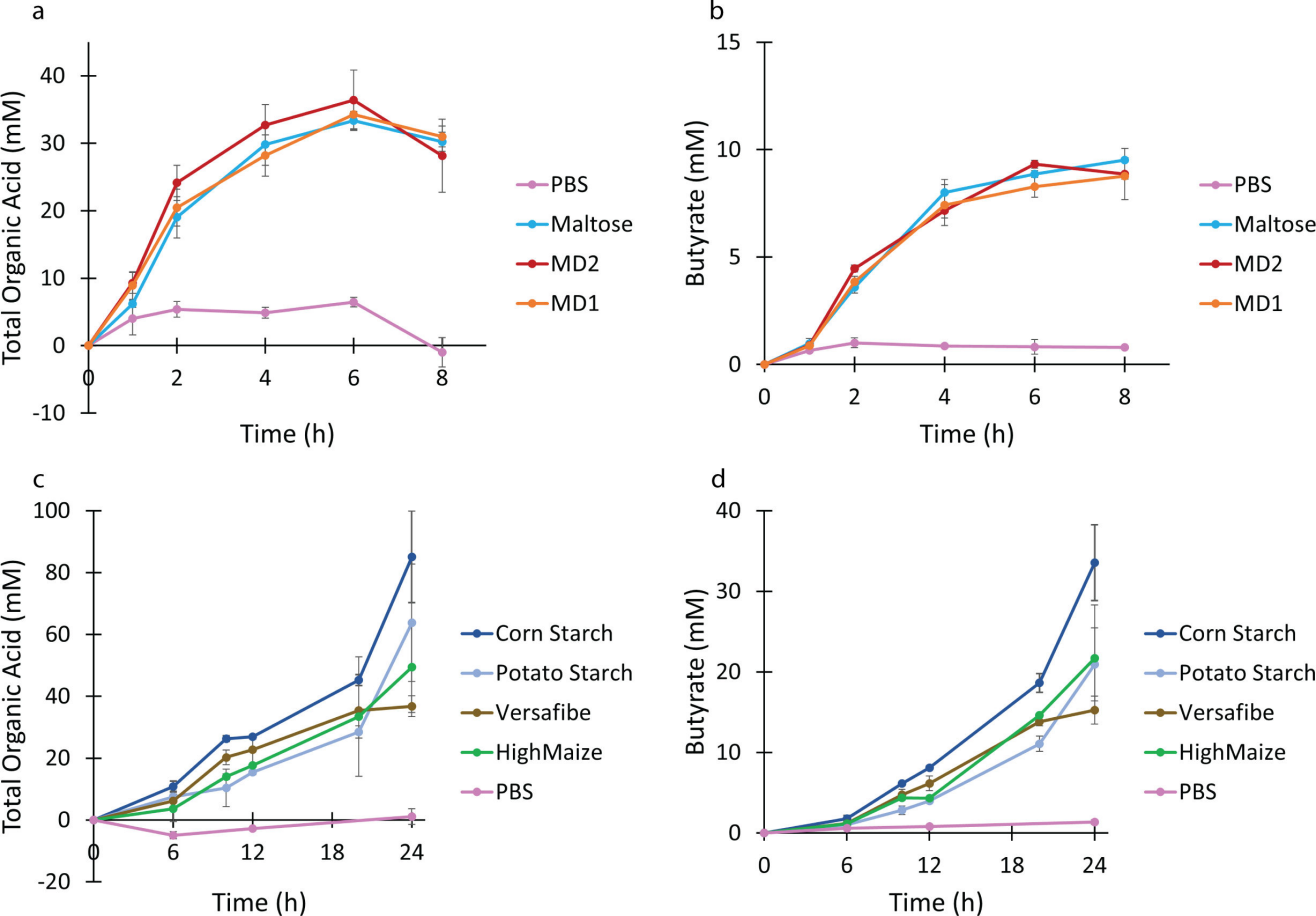
Production of organic acids by *Clostridium butyricum* during growth on soluble and insoluble substrates. Panels a and b represent the production of total organic acids and butyrate, respectively, during growth on soluble substrates. Panels c and d represent the production of total organic acids and butyrate, respectively, during growth on insoluble substrates. All growths were carried out in an anaerobic chamber at 37°C in RUM media supplemented with the indicated substrate. Organic acids were measured by HPLC and total organic acids (a and c) were the sum of formate, acetate, butyrate, and lactate detected in the samples. All experiments were conducted in triplicate with error bars indicating the standard deviation. MD1, dextrose equivalent 16.5–19.5,; MD2, dextrose equivalent 4.0–7.0; HiMaize, Hi-MAIZE 260; and Versafibe, VERSAFIBE 2470.

To investigate the starch utilization potential of *C. butyricum*, we first performed a series of growth experiments. We performed *in vitro* fermentation of various soluble (maltose, maltodextrins DE 4.0–7.0, and DE 16.5–19.5) and insoluble (corn starch, CS; non-RS; potato starch, PS; type 2 RS; HI-MAIZE 260 cornstarch, HM; type 2 RS; VERSAFIBE 2470, VF; and type 4 RS) substrates to compare the ability of *C. butyricum* to utilize them for growth as compared to the RUM media supplemented only with phosphate-buffered saline (PBS) (Fig. 1). Given the challenges associated with monitoring growth on insoluble substrates, we chose to use organic acid production by *C. butyricum* as a proxy for growth (Fig. 1). *C. butyricum* produced a mixture of formate, acetate, butyrate, and lactate during growth on all starch substrates, though there was minimal production detected when the media were supplemented with PBS instead of carbohydrate substrate. Growth of *C. butyricum* on the soluble substrates was rapid, with the bacterium exhausting the available substrates in about 6 h (Fig. 1a and b). There were no significant differences in either the total organic acid production or the butyrate production between the soluble substrates. Growth on the insoluble substrates was somewhat slower, not reaching a plateau of organic acid production by the 24-h time point, though the amount of organic acid production was substantially higher than for the soluble substrates (Fig. 1c and d). There was likewise no difference in the rate of organic acid production between the insoluble substrates. This demonstrates that *C. butyricum* is capable of using a variety of starches, including several RS substrates.

#### Starch adherence

**Fig 2:**
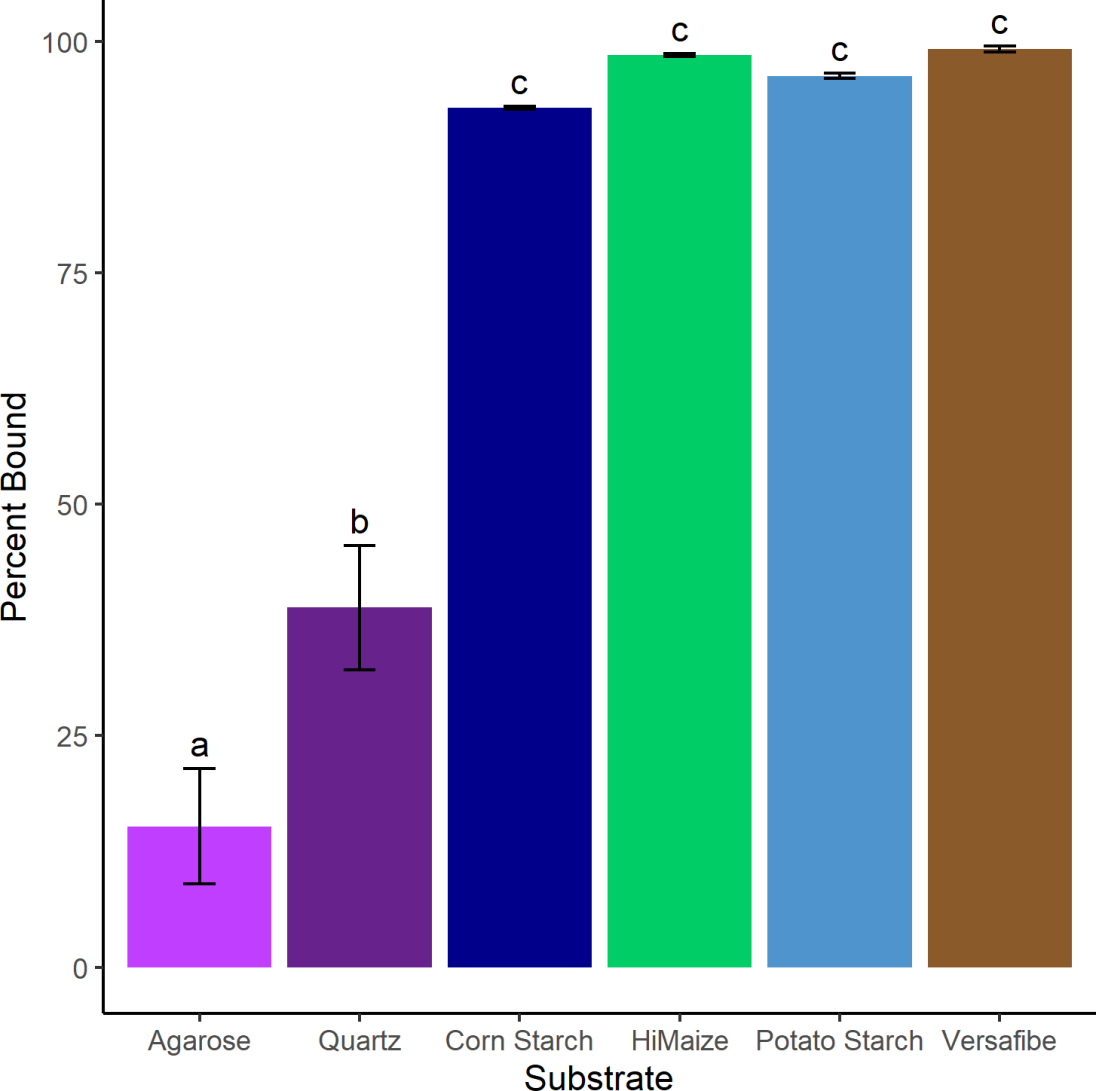
Binding of *Clostridium butyricum* to insoluble substrates. Cells were incubated with the indicated insoluble substrate, which was then removed by low-speed centrifugation, and the supernatant was plated. The data are presented as percent bound to the insoluble substrate (the percent reduction in cell count relative to a no substrate control). All experiments were conducted in triplicate with error bars representing the standard deviation. Means were compared by one-way ANOVA with Tukey’s *post hoc* test. HiMaize, Hi-MAIZE 260 and Versafibe, VERSAFIBE 2470.

Within the human gut, there is intense competition for substrates and binding tightly to substrates can provide an advantage, so we tested if *C. butyricum* can bind tightly and specifically to these RS substrates. The results of the adherence assay (Fig. 2) indicated that *C. butyricum* can bind quite well to multiple types of starch granules, with 99% ± 0.3% bound to VF, 98% ± 0.2% bound to HM, 96% ± 0.3% bound to PS, and 92% ± 0.2% bound to CS, with no significant differences between them. This was in contrast to the general control (quartz sand, 39% bound) and a non-utilized carbohydrate control (agarose; 15% bound). Given the ability of *C. butyricum* to adhere strongly to starch granules, this suggests that it could compete strongly for these substrates within the gut environment.

#### Scanning electron microscopy of RS granules after simulated digestion and post fermentation

**Fig 3:**
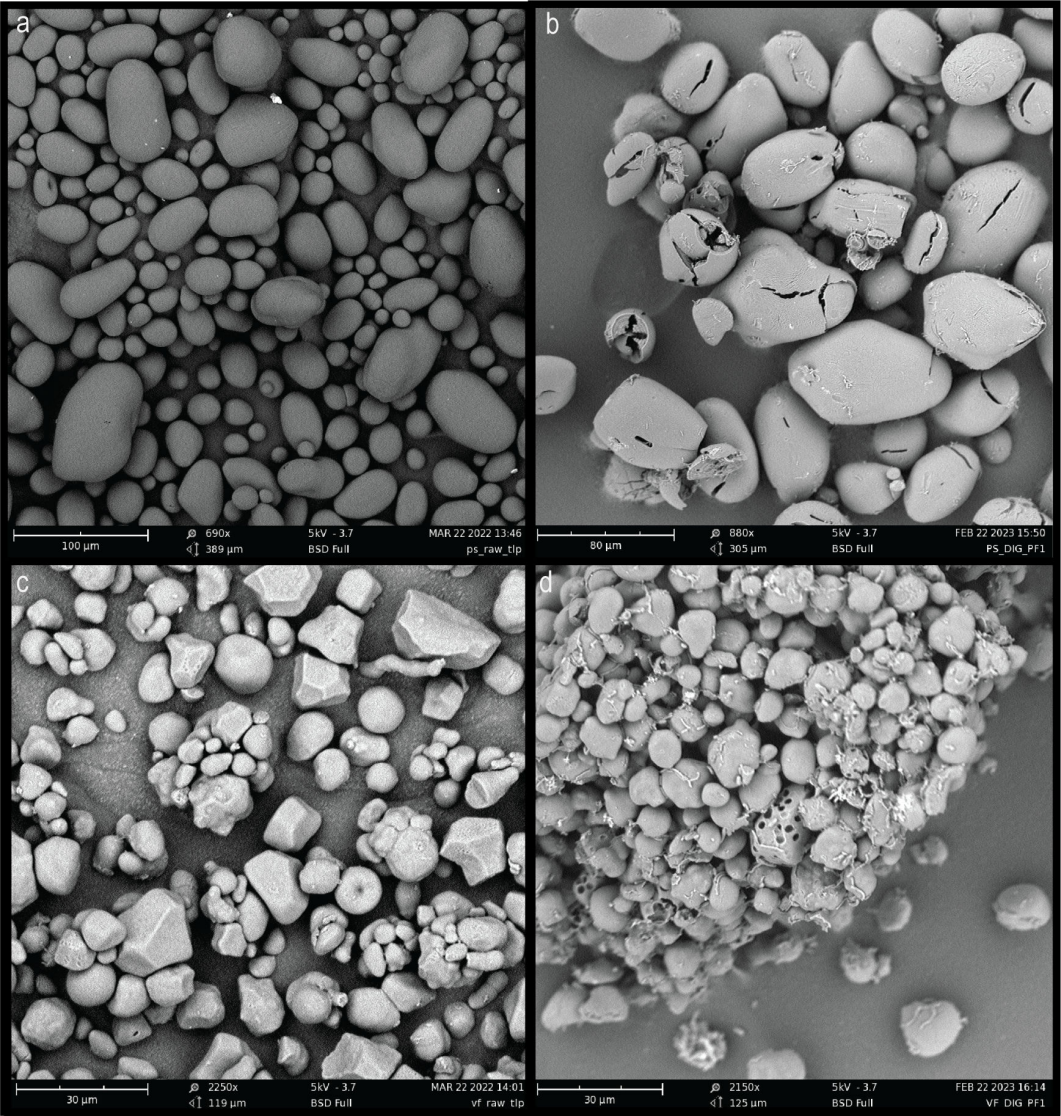
Scanning electron microscopy images of starch granules. (a) Potato starch granules post-simulated digestion and (b) after *Clostridium butyricum* fermentation for 24 h at 37°C, with no shaking. (c) VERSAFIBE 2470 granules post-simulated digestion and (d) after *C. butyricum* fermentation under the conditions described above. The voltage employed was 5 kV, and magnification varied based on starch granule size. For the potato starch granules, which are larger in size, magnifications of 690× (pre-fermentation, panel a) and 880× (post-fermentation, panel b) were used. For the VERSAFIBE 2470 granules, magnifications of 2,250× (pre-fermentation, panel c) and 2,150× (post-fermentation, panel d) were used.

To further investigate the digestion of RS by *C. butyricum*, SEM was performed on granules at the beginning and end of growth experiments (Fig. 3). As seen in Fig. 3a and c, the simulated intestinal digestion prior to the growth experiment did not noticeably impact the structure of the starch granules. Figure 3b and d represents PS and VF, respectively, after 24 h of fermentation by *C. butyricum*. These images indicate that there is a difference in the degradation pattern between these two substrates, with the organism forming cracks in the exterior of the PS granule to reach the interior portion, as can be seen in Fig. 3b. This contrasts with the degradation patterns exhibited toward the other insoluble substrates in this study, which similarly exhibited pore formation as seen on the VF granules in Fig. 3d.

### Characterization of the enzymes

#### Enzyme identification and conservation

**Fig 4:**
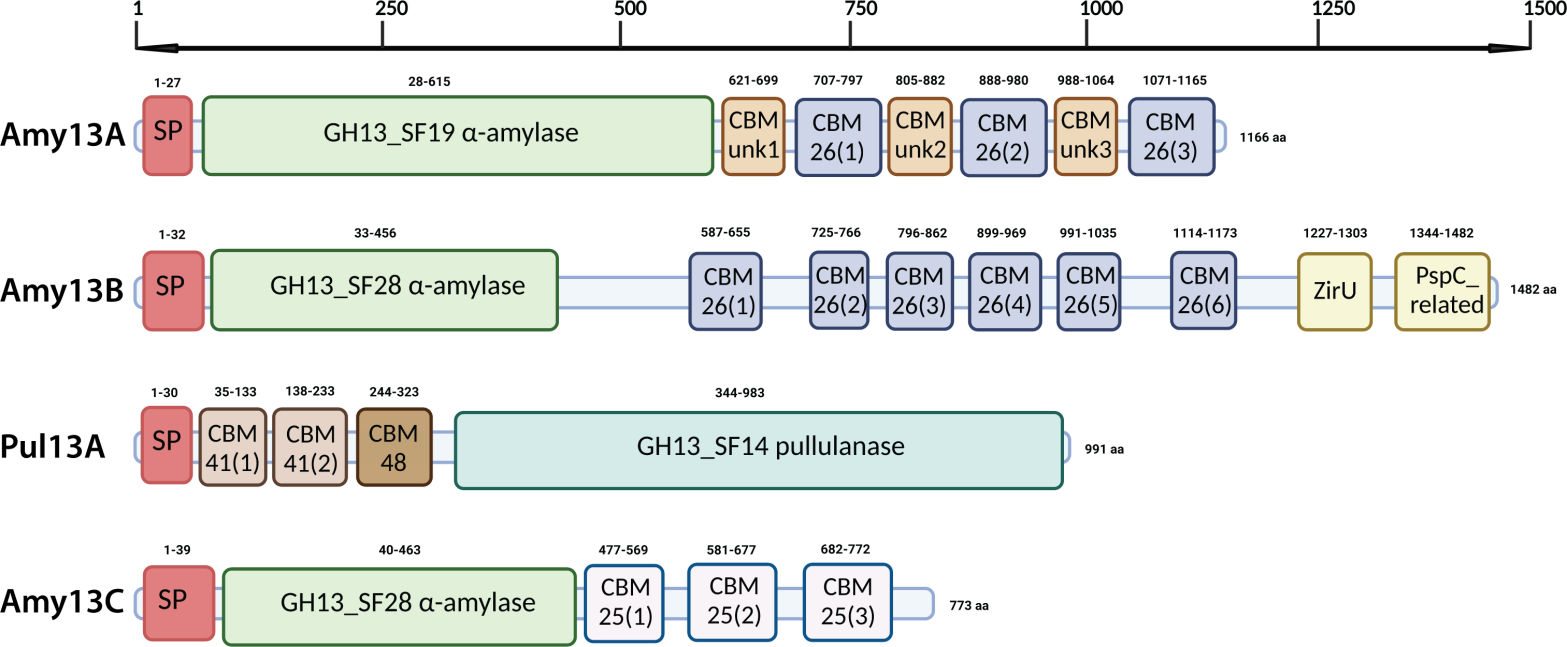
Major predicted extracellular amylases and pullulanase of *C. butyricum* Prazmowski. Domain organization shown is predicted using a combination of the NCBI conserved domain database, the CAZy database, and AlphaFold-generated structures (https://alphafold.ebi.ac.uk/). Signal peptides (SP) are predicted by SignalP-6.0 (https://dtu.biolib.com/SignalP-6). All domains are scaled according to amino acid length. Protein nomenclature is as described in Table S2. Created with BioRender.com.

To identify the enzymes responsible for starch digestion in *C. butyricum* Prazmowski, the genome was searched for GH13 family enzymes that contained a predicted signal peptide, which resulted in four candidate enzymes. One is a member of GH13 subfamily 19 (GH13_SF19; Amy13A; UniProt: A0A7G5NSF6; GenBank: QMW89799.1) and two are members of GH13 subfamily 28 (GH13_SF28; Amy13B; UniProt: A0A7G5NT35; GenBank: QMW90028.1 and Amy13C; UniProt: A0A7G5NWM1; GenBank: QMW91264.1) as predicted extracellular α-amylases (EC 3.2.1.1). There is also a member of GH13 subfamily 14 (GH13_14; Pul13A; UniProt: A0A7G5NUT4; GenBank: QMW90627.1) as a predicted extracellular pullulanase (EC 3.2.1.41). Each of these enzymes possesses multiple CBMs across families CBM25 and CBM26, in the case of the α-amylases, or CBM41 and CBM48, in the case of the pullulanase (Fig. 4). These four enzymes were found to be highly conserved across the other *C. butyricum* strains listed in the CAZy database, with similar systems found in 11 of the 15 strains (Table S2). The conservation of these proteins was confirmed by BLASTp of these proteins and the resulting distance trees. Interestingly, the four divergent strains have a similar number of GH13s and CBM48s, and while they do possess a GH13_SF14 pullulanase, it does not have a CBM41 nor is it predicted to have a signal peptide, indicating it is doing its work intracellularly. Along with the four extracellular enzymes, there are 15 other GH13 family enzymes annotated in the genome of *C. butyricum* Prazmowski in the CAZy database, all of which are predicted to have a cytoplasmic location (Table S3). Several of these enzymes are predicted to have glucosidase activity on α(1, 4) and/or α(1, 6)-linked maltooligosaccharides and disaccharides to produce maltose and glucose. It is likely that the cytoplasmic GH enzymes contribute to the final processing of imported oligosaccharides or participate in the synthesis and breakdown of internal glycogen stores.

#### Zymogram analysis

**Fig 5:**
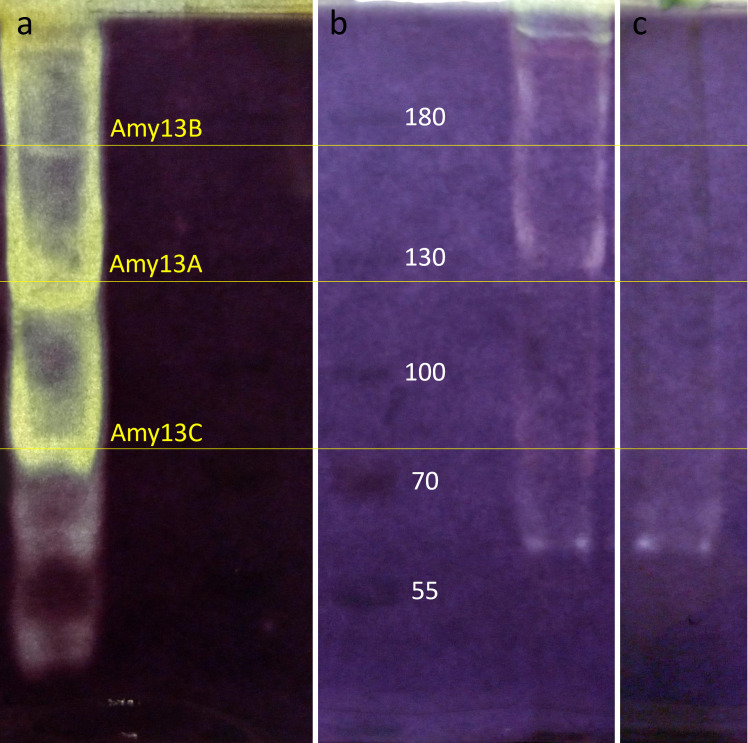
Zymogram analysis of recombinant and native *Clostridium butyricum* starch degrading enzymes. Zymograms were conducted by performing SDS-PAGE with 0.2% amylopectin incorporated into 7.5% polyacrylamide gels and then renaturing the gels in 50 mM Tris-HCl pH 6.8, 2 mM DTT, 1 mM EDTA, and 5 mM CaCl_2_ overnight at 4°C. After renaturation, the gels were incubated for 4 h at 37°C and then stained with iodine. (a) The first lane is a mixture of Amy13A, Amy13B, Amy13C, and Pul13A that have been recombinantly expressed in *E. coli*, purified, and then combined before loading into the gel. This is followed by a blank lane and then the PageRuler pre-stained ladder. (b) The first lane is the PageRuler pre-stained ladder, followed by a blank lane, and then the supernatant of *C. butyricum* cells grown in RUM media with maltose for 6 h at 37°C in an anaerobic chamber. (c) Supernatant of *C. butyricum* cells grown in RUM media with glucose for 6 h at 37°C in an anaerobic chamber. Note that panels b and c are from the same gel with the intervening lanes removed for ease of comparison.

To determine if the predicted extracellular pullulanase and α-amylases were produced during growth on starch, *C. butyricum* was grown on both glucose and maltose, the supernatants were isolated and then assayed in an amylopectin zymogram (Fig. 5). For comparison purposes, recombinantly produced Amy13A, Amy13B, Amy13C, and Pul13A were also subjected to zymogram analysis (Fig. 5a). For each of Amy13A (129 kDa), Amy13B (166 kDa), and Amy13C (84 kDa), zones of clearing appearing at the approximate predicted molecular weight could be seen for both the recombinantly produced protein (Fig. 5a) and from *C. butyricum* culture when grown on maltose (Fig. 5b) but not from *C. butyricum* culture when grown on glucose (Fig. 5c). Bands from the native system ran slightly higher than the recombinant proteins, perhaps indicating some type of post-translational modification for the native enzymes. Pul13A (113 kDa) was not visible in either of the zymograms, perhaps because pullulanase activity is not as easily detected with this substrate. Two lower molecular weight bands present in the recombinantly produced proteins probably represent still active proteolytic digestion products. There is a low molecular weight band in the *C. butyricum* supernatant, present in both the maltose-grown and glucose-grown cells, though its identity is uncertain given that *C. butyricum* has a number of GH13 enzymes in this size range within its genome (Table S3). There is also a high molecular weight band found in both the recombinant protein mixture and the maltose-grown *C. butyricum* supernatant. This is likely to be proteins that were not fully unfolded during the milder than typical incubation in loading buffer (performed at 60°C) whose progress through the gel was then slowed through interaction with the incorporated amylopectin as is typically seen in affinity electrophoresis experiments (31). These results suggest that maltose serves as an inducer of *C. butyricum*’s starch digestion system and that Amy13A, Amy13B, and Amy13C are the key α-amylases of that system. The involvement of Pul13A remains less certain but given that is the only pullulanase in the genome of *C. butyricum* that possesses a predicted signal peptide, it is likely to be involved as well.

#### Enzyme characterization with soluble substrates

**Fig 6:**
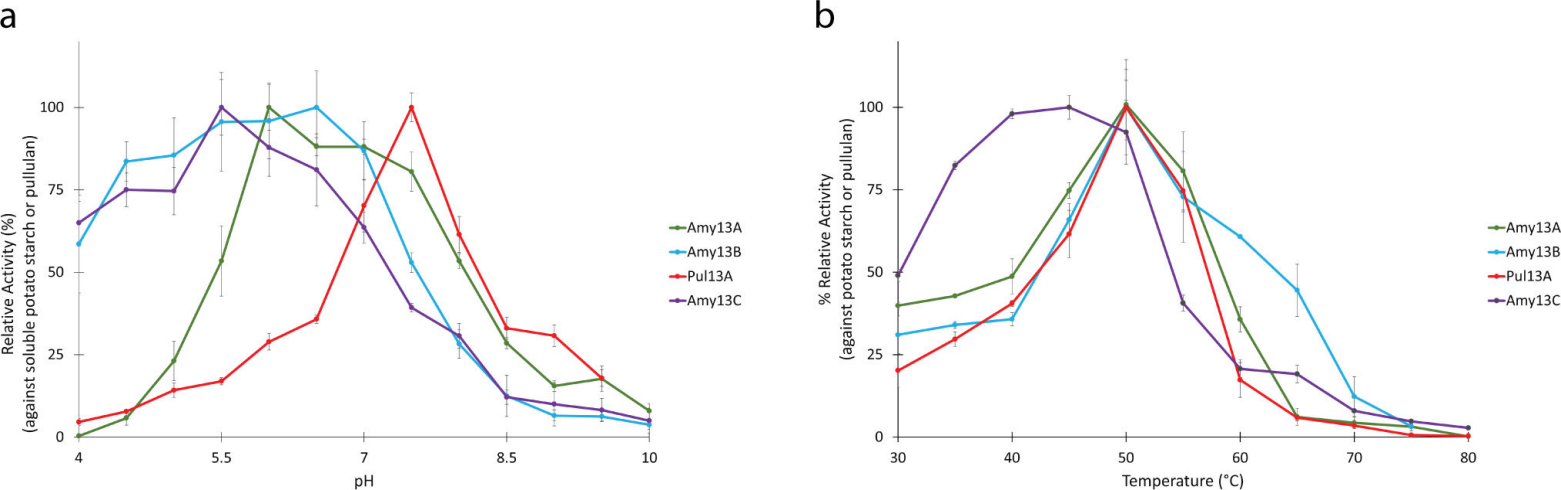
Determination of pH and temperature optimum for the four recombinant *C. butyricum* enzymes. (a) pH versus the percent relative activity determined using the model substrate of soluble potato starch (Amy13A, Amy13B, and Amy13C) or pullulan (Pul13A). The pH optimum varied for the enzymes; however, pH 7 was chosen for further experiments as it represented both a biologically relevant pH and accounted for the best representation of activity for all the enzymes. (b) Temperature (°C) versus the percent relative activity determined using the model substrates as described above. There was some variance in the optimum temperature from 45°C to 50°C; however, 37°C was used for all enzymes as it is most representative of a biological system.

To confirm the predicted activities of these enzymes and to investigate their characteristics, each of the four enzymes was cloned for recombinant production in *E. coli*. Each enzyme was characterized in terms of its pH and temperature optimum, and it was found that the pH optimum varied between pH 5.5 and pH 7.5, while the temperature optimum was 50°C for all enzymes except for Amy13C, which was closer to 45°C (Fig. 6). Moving forward, pH 7.0 and 37°C were used in the characterization experiments as these conditions are representative of biological conditions in the human gut and it was shown that the enzymes are highly active under these conditions.

Enzymes were characterized against the soluble substrates dextran, α-, β-, and γ-cyclodextrins, glycogen, amylose, amylopectin, soluble potato starch, and pullulan (Table 1). Results showed that there were several differences in soluble carbohydrate usage between the enzymes. Both Amy13A and Amy13B showed some activity toward β-cyclodextrin while the other enzymes did not, while only Amy13A showed any activity toward γ-cyclodextrin. Both Amy13A and Pul13A were active against glycogen, while all of the putative α-amylases were active against amylose. All four enzymes exhibited activity toward amylopectin and soluble starch. As expected Pul13A had a strong activity toward pullulan, but there were also low but detectable levels of activity seen in Amy13A and Amy13B, but not Amy13C. Overall, the results were strongly supportive of the identification of Pul13A as a pullulanase, while Amy13A, Amy13B, and Amy13C can be classified as α-amylases, though with different substrate preferences.

**TABLE 1:** Activity (nmol min^−1^ mg^−1^) ± standard deviation and percent maximal activity of *Clostridium butyricum* enzymes toward soluble substrates (each at 0.03%)^*a*^^,^^*b*^

Substrate	Amy13A	Amy13B	Pul13A	Amy13C
Dextran	–	–	–	–
α-Cyclodextrin	–	–	–	–
β-Cyclodextrin	202 ± 32 (11.6%)	656 ± 73 (11.2%)	–	–
γ-Cyclodextrin	91 ± 21 (5.2%)	–	–	–
Glycogen	1,170 ± 103 (67.1%)	–	136 ± 57 (8.5%)	–
Amylose	1,680 ± 245 (96.4%)	2,880 ± 220 (49.3%)	–	672 ± 79 (88.0%)
Amylopectin	1,740 ± 108 (100%)	4,440 ± 1,290 (76.1%)	955 ± 228 (59.9%)	428 ± 93 (56.0%)
Pullulan	5 ± 1 (0.3%)	6 ± 1 (0.1%)	1,590 ± 45 (100%)	–
Soluble starch	1,460 ± 57 (83.9%)	5,840 ± 378 (100%)	474 ± 111 (29.7%)	764 ± 79 (100%)

^*a*^ Relative to the best substrate for that enzyme, indicated by 100% activity.

^*b*^ - Indicates that activity was not detected for that enzyme/substrate combination.

#### Characterization of enzymes with insoluble starch granules

**Fig 7:**
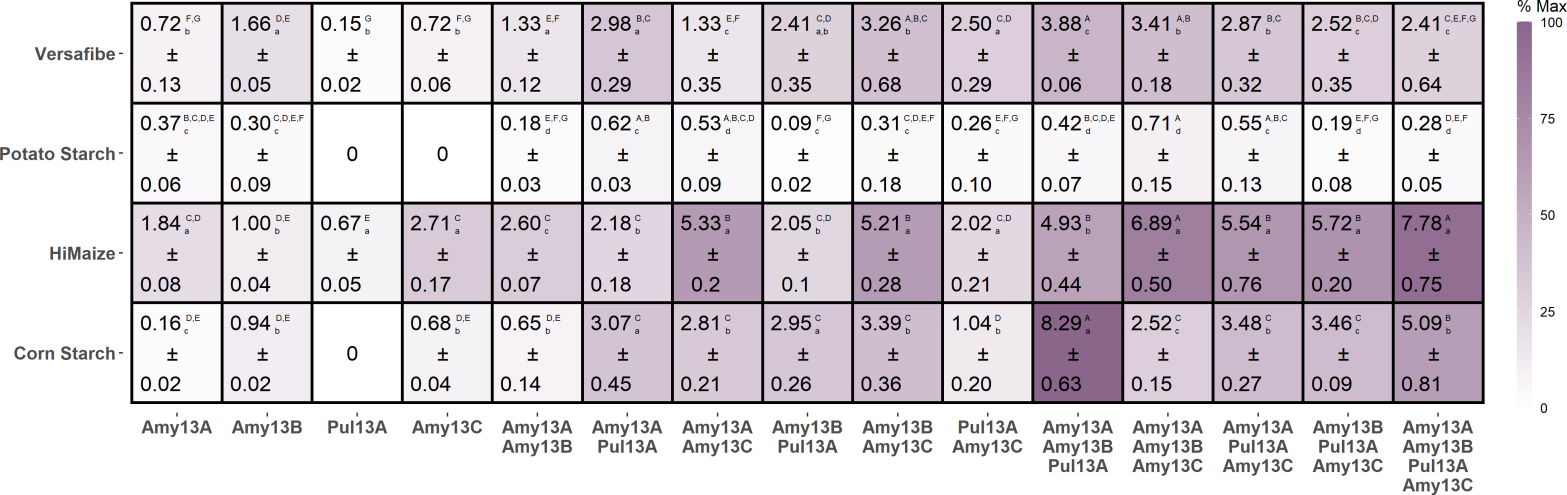
Synergy between *C. butyricum* enzymes during digestion of starch. Values are reaction velocities (nmol product h^−1^) of the indicated enzyme or enzyme combination. Background color intensity indicates the percentage of the maximal activity produced across all experiments. For each assay, 5 U (nmol min^−1^) of soluble substrate activity (measured toward soluble potato starch for amylases and pullulan for the pullulanase) was added. For example, in single-enzyme assays, 5 U of that enzyme was added, but for the four-enzyme assay, it was 1.25 U of each to avoid over-saturation of available binding sites. The experiments were carried out in triplicate at 37°C for 12 h and activity was measured via reducing sugar production. One-way ANOVA was performed to compare the means with a Tukey’s HSD *post hoc* test (*α* = 0.05) to determine the significance levels of each substrate (columns, significance grouping denoted by upper-case letters) and each enzyme(s) (rows, significance grouping denoted by lower-case letters). Due to the number of comparisons, the *P*-values can be found in File S2.

To further understand the capabilities of these enzymes, their activities toward CS and RS substrates were investigated and are presented in Fig. 7. Note that the amount of each enzyme added was normalized based on activity toward soluble potato starch for the amylases and pullulan for the pullulanase Pul13A, and for each reaction, the same total units of soluble substrate activity were added. This approach ensures that detectable activity levels of each enzyme are included in the assays, while also avoiding oversaturating the available binding sites on the starch granules. Thus, in this setup, higher activity seen in combinations relative to the highest activity seen in the component individual enzymes represents synergy. The combination of Amy13A + Amy13B + Pul13A with CS exhibited the highest activity overall; however, different combinations were optimal for each of the substrates tested. As was seen for CS, the Amy13A + Amy13B + Pul13A combination was best for VF, though it only had about half the activity as displayed toward CS. For HM, the combination of all enzymes was best, nearly reaching the maximal activity seen in the assay. For PS, the Amy13A + Amy13B + Amy13C combination was the best, though this reached only 10% of the maximum activity seen toward CS. Among the individual enzymes, Amy13C had its best activity toward HM and the two-enzyme and three-enzyme combinations including Amy13C outperformed the other combinations with this substrate. In contrast, Amy13B had its best activity toward VF, and the top three combinations with this substrate all included Amy13B. While Amy13A exhibited its best activity toward HM, it had the best activity of any of the four enzymes toward PS, and the top five combinations with this substrate all included Amy13A. Pul13A only displayed measurable activity toward HM and VF; however, it was an important contributor to synergy in many cases. For instance, with CS, the activities (nmol reducing sugar h^−1^) of Amy13A (0.16 ± 0.01) and Amy13B (0.94 ± 0.02) were dramatically improved when paired with Pul13A (Amy13A + Pul13A, 2.81 ± 0.21; Amy13B + Pul13A, 2.95 ± 0.26; Amy13A:Amy13A + Pul13A, *P* = 0.00004; Amy13B:Amy13B + Pul13A, *P* = 0.01713), and the combination of all three with CS was the highest activity measured with a mean of 8.29 nmol h^−1^. These results demonstrate that the *C. butyricum* enzymes can degrade RS in a synergistic manner, though with individual enzymes displaying differing preferences and levels of importance for the digestion of the various RS substrates.

#### Characterization of product profile

**Fig 8:**
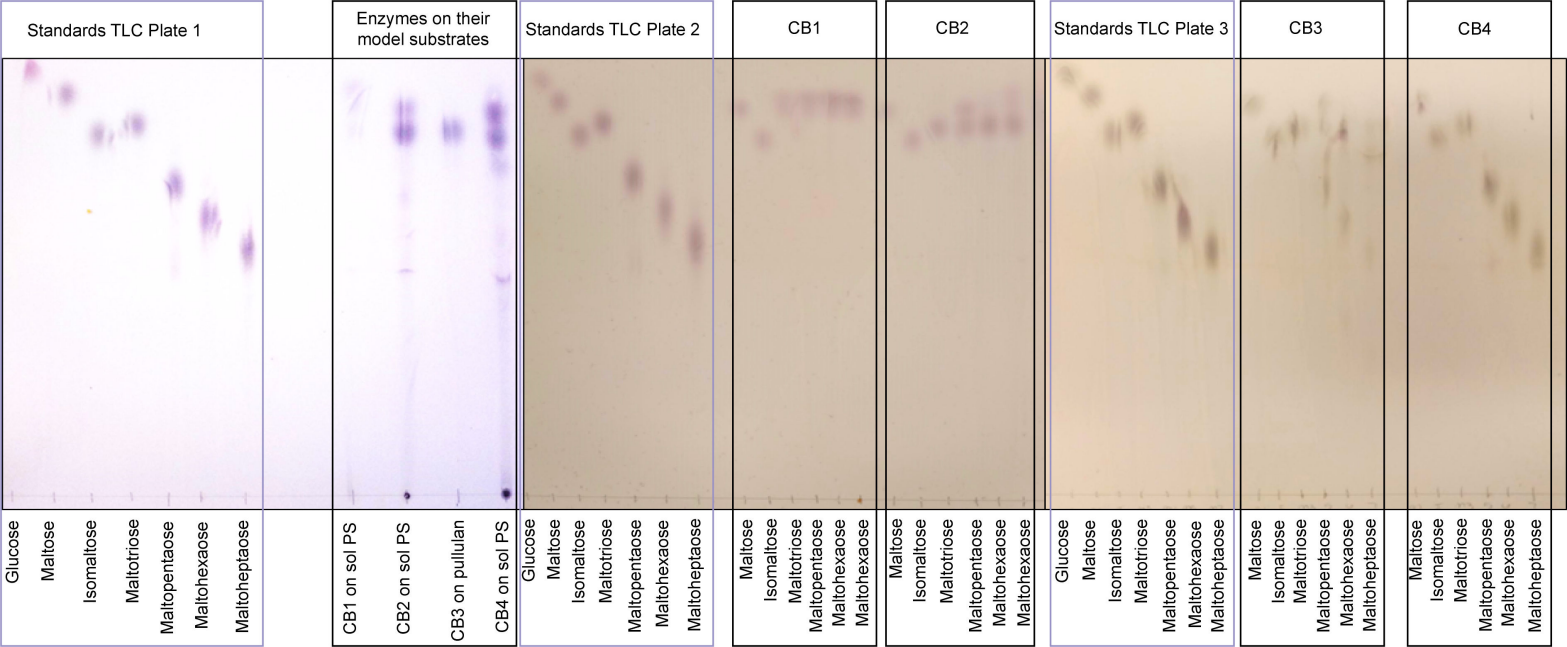
Thin layer chromatography analysis of product profiles. In all cases, the standards used were glucose, maltose, isomaltose, maltotriose, maltopentaose, maltohexaose, and maltoheptaose. From left to right: plate 1, the products generated by the enzymes against their model substrates (Amy13A, 2, and 4 against soluble potato starch and Pul13A against pullulan). Plate 2, the products generated by Amy13A and Amy13B against maltose and isomaltose (negative controls), maltotriose, maltopentaose, maltohexaose, and maltoheptaose. Plate 3, the products generated by Pul13A and Amy13C against maltose and isomaltose (negative controls), maltotriose, maltopentaose, maltohexaose, and maltoheptaose. The enzyme reactions were performed at 37°C for 16 h with a substrate concentration of 1.5% (wt/vol), the mobile phase was prepared with acetonitrile, ethyl acetate, 2-propanol, and deionized water. The staining solution consisted of sulfuric acid and methanol, with N-(1-naphthyl)ethylenediamine dihydrochloride dissolved to produce the final solution. TLC silica gel 60 F_254_ plates were used for separation.

The product profiles of the *C. butyricum* enzymes were examined by thin-layer chromatography (TLC), using glucose, maltose, isomaltose, maltotriose, maltopentaose, maltohexaose, and maltoheptaose as standards. Plate 1 (Fig. 8) shows the products formed when the enzymes were tested against their model substrates, which were soluble PS in the cases of Amy13A, Amy13B, and Amy13C and pullulan in the case of the putative pullulanase Pul13A. Maltose and maltotriose were produced by the putative amylases Amy13A, Amy13B, and Amy13C. Pul13A produced maltotriose from pullulan, which is to be expected from a Type I pullulanase (32). Plates 2 and 3 show the products formed when the maltose, isomaltose, and the maltooligosaccharide standards were treated with the isolated enzymes. None of the enzymes tested exhibited any ability to degrade maltose or isomaltose to glucose. Amy13A produced maltose from the maltooligosaccharides, while treatment of these substrates with Amy13B indicated that it is unable to cleave maltotriose to maltose but can do so for the longer chains, yielding maltose and maltotriose. Interestingly, treatment of the maltooligosaccharides with Amy13C indicates that it does not have the ability to further degrade these shorter oligosaccharides, or at least not under the conditions tested.

## DISCUSSION

### Comparison of *Clostridium butyricum* to other resistant starchdegrading bacteria

The starch-degrading enzyme system of *C. butyricum* shares many commonalities with those from *R. bromii* and *B. adolescentis*, including the presence of α-amylases from the GH13_28 and GH13_19 subfamilies, a pullulanase from the GH13_14 subfamily, and CBMs from families CBM25 and CBM26, which are encoded within their multi-domain α-amylases (Fig. S1). While RS-degrading organisms encode a diversity of enzymes and non-catalytic modules to aid in the digestion of RS, many of these are shared with non-RS-degrading organisms as well. However, some factors such as the presence of the GH13_28 subfamily or the CBM74 family are almost exclusive to RS-degrading organisms (Fig. S2). Modules of the CBM74 family have been identified as a driver for RS degradation and, further, have been shown to play an important role in granule pore formation during the fermentation process (33). Furthermore, the structure of a representative of this family from *R. bromii* was recently solved and demonstrates an architecture and binding capacity much different than other starch-binding CBM families, providing perhaps a unique advantage for RS degraders (34). However, this CBM family appears to be absent from the *C. butyricum* genome, and so our results would indicate that while the CBM74 may be playing an important role in RS degradation, it is not an absolute requirement. Additionally, while the characterization of the other RS-degrading organisms indicates that physically linked enzymes such as the amylosome of *R. bromii* or the bifunctional amylase-pullulanase of *B. adolescentis* may be important for their degradation capacity, this is also absent from *C. butyricum* (35–37). In Fig. 4, it can be seen that the *C. butyricum* α-amylases possess multiple CBM26 and CBM25 domains, as seen in other RS degraders, but also some unknown CBM-like domains in Amy13A and domains at the end of Amy13B that are of unknown function. Intriguingly, these unknown potential CBM families seem to exist in tandem repeats with the CBM26s of Amy13A. As seen in Fig. S3, the three unknown CBMs cluster together, separate from the CBM26s found in both Amy13A and Amy13B, which form their own distinct cluster; nor is it likely that these are from the closely related family CBM25 based on the results of the phylogenetic comparisons. Using the AlphaFold predictions of the structure of these domains and searching against the Protein Data Bank (https://www.rcsb.org/) using the DALI server (http://ekhidna2.biocenter.helsinki.fi/dali/) did not reveal structural homology to any known CBM families (38). Taken together, these results suggest that these could represent CBMs from a novel family and thus these deserve closer investigation of their binding functionality. Overall, it appears that there may not be one key component that is absolutely required for RS degradation and utilization by gut microorganisms, but rather there are multiple viable paths to RS utilization. The enzyme system of *C. butyricum* is perhaps the simplest of the RS degraders described to date; however, even here there are unique features that require further investigation to elucidate their importance.

### Synergy in *Clostridium butyricum* enzymes

To better understand how *C. butyricum* can degrade RS with its repertoire of enzymes, we investigated all possible combinations of these enzymes against corn starch and three sources of RS. We found significant synergy as compared to the best individual enzyme for each substrate, the best combination exhibited at least double the activity (Fig. 7). Furthermore, the best combination was different for every starch source tested, providing a rationale for having multiple, seemingly redundant enzyme activities within the same enzyme system. This type of synergy and functional specialization seems likely to be an important theme for RS-degrading organisms, given the similar diversity of α-amylases seen in *R. bromii* (35) and *B. adolescentis* (21). Degradative synergy has been studied in a number of other carbohydrates, most prominently cellulose. In this case, several classes of synergism have been identified, typically between enzymes with different types of activity (39). This includes endo-exo synergism between endoglucanases (hydrolyzing in the middle of chains; endo-acting) and cellobiohydrolases (releasing the disaccharide cellobiose from chain ends; exo-acting) (40). In this case, the enzymes differ in both their site of action (the middle of chains versus chain ends) and in their product profiles, generally a random assortment of oligosaccharides for endoglucanases and specifically cellobiose for cellobiohydrolases (41). This is akin to the combination of α-amylases (endo-acting) and β-amylases (exo-acting) used by plants for starch hydrolysis during germination (42). However, like RS-degrading organisms encoding multiple α-amylases, cellulose-degrading organisms typically encode multiple endoglucanases, suggesting that synergy between these enzymes may also be important (43, 44). This could arise through differences in binding sites driven by differences in the non-catalytic components of the enzymes such as CBMs or it may be related to differences in product profiles as seen in Fig. 8 for the *C. butyricum* α-amylases. The α-amylases of *C. butyricum* differ far more in their non-catalytic domain regions than they do within the GH13 domains themselves, suggesting that these differences in the CBMs present may drive the localization of these enzymes to specific structural motifs present in starch granules, thus allowing for synergy. While this endo-endo synergy is not often studied, it has been reported previously for a pair of α-amylases (45). Additionally, unlike in cellulose, there are two different linkages present between the glucose molecules, the α1,4 linkages of the backbone and the α1,6 linkages of the branches. This presents another opportunity for synergism and indeed the Pul13A pullulanase (active on the α1,6 linkages), though it displayed little activity toward the starch substrates on its own, was typically part of the most synergistic combinations (Fig. 7). Thus, although *C. butyricum* has a simpler starch-degrading system than other RS-degrading organisms, it has a set of enzymes that can work together efficiently.

### Model of resistant starch utilization by *Clostridium butyricum*

According to the rates of organic acid production (Fig. 1), all the starches were fermented similarly; however, interestingly, this is not reflected in the enzyme assay results where PS was a much poorer substrate, digested at one-tenth the rate of CS (Fig. 7). This contrasts with what was seen in *Eubacterium rectale* where its primary amylase had poor activity toward PS reflected in poor growth of the organism on that substrate (46). To grow on starch, an organism must bind to the granules, enzymatically digest the starch, transport the digestion products into the cell, and utilize them for growth. It is evident from Fig. 2 that *C. butyricum* can efficiently bind to starch, but the mechanism remains unclear. The four extracellular enzymes all contain starch-specific CBMs; however, their signal sequences are predicted to be cleaved and there are no predicted sortase motifs to localize them to the cell wall. It is possible that the domains of unknown function found in Amy13A and Amy13B (Fig. 4) might play a cell surface-anchoring role; however, they do not bear any sequence similarity to other binding domains that are known to play this role. This suggests that there may be other non-enzymatic factors at play in the digestion of starch by *C. butyricum. R. bromii* encodes several non-enzymatic components as part of its amylosome, including the recently described Sas20, which seems to play an important role in starch binding (47). Similarly, the Sus system of *Bacteroides thetaiotaomicron* includes the non-enzymatic proteins SusE and SusF, which are important starch-binding proteins but may also facilitate the uptake of starch degradation products (48, 49). Thus, it is possible that *C. butyricum* has additional non-enzymatic factors that are important for its RS-degrading ability, though it should be noted that there are no homologs of Sas20, SusE, or SusF present in its genome; so, identification of these factors will require further study. However, a further suggestion that such factors may exist comes from the SEM results for growth on potato starch in comparison to growth on corn starch derivatives (Fig. 3) where an alternate mode of digestion was found, cracking versus pitting, which has been previously noted for amylases in other systems (50, 51). While factors that drive binding to the starch granules require further investigation, transporters for starch digestion products are more readily found within the *C. butyricum* genome. Two glucose-specific Enzyme IIA components of PTS transporters are annotated within the *C. butyricum* genome (WP_002581229 and WP_002583131.1) as well as a maltose-specific solute-binding protein of an ABC transporter (WP_035765177.1). While there are no annotated maltooligosaccharide transporters within the *C. butyricum* genome, a BLAST search using the solute-binding protein EUR_01830 from the *Eubacterium rectale* maltooligosaccharide-specific ABC transporter (52) reveals a strong match (WP_003406799.1; *E*-value 7 × 10^−97^). Thus, it appears that *C. butyricum* has all of the required transport machinery to support growth on resistant starch.

### Butyrate production by *Clostridium butyricum* during growth on starch

Butyrate is recognized as an important metabolite in sustaining the physical and mental well-being of most humans, even improving certain disease states (11); but attempts to promote butyrate formation through RS supplementation have yielded mixed results (53). There is a lot of evidence that the individual’s native microbiome plays an integral role in its ability both to degrade the intact granules and then to produce butyrate from the metabolic by-products (53, 54), which relies on two separate groups of bacteria, primary RS degraders and butyrate producers, interacting through a cross-feeding mechanism (6, 55, 56). However, *C. butyricum* combines both functionalities into a single organism (Fig. 1), which should lead to a more universal butyrate increase when *C. butyricum* is the primary RS degrader present. While it has been shown here and previously (23, 57, 58) that *C. butyricum* can utilize some types of RS and is a butyrate-producing organism, there is not much evidence to indicate that there is a noticeable increase in its relative abundance in response to RS dietary intervention studies, although it was shown to increase in human flora-associated rats in response to “CrystaLean” (a retrograded, amylose starch) supplementation (59). It could be that it is present in adult microbiomes in such low abundance to be overlooked by algorithm cutoffs. One could speculate that in a dynamic community of microorganisms and myriad other undigested foodstuffs and metabolites, RS is simply not the preferred means of energy generation for *C. butyricum* or that it cannot outcompete other RS-degrading bacteria. Although it may not be naturally present at high levels in humans, *C. butyricum* may be a strong candidate for use as a probiotic. Supplementation with *C. butyricum* MIYAIRI 588 has been shown to improve disease states (26) and overall health (60). It has been employed as a probiotic in Asian countries since the 1950s, but its modes of action are just now being understood (25, 26). There is still much we do not know, but many of its positive effects seem to be linked to the pleiotropic role butyrate plays throughout the length of the gastrointestinal tract (60). Further human studies using *C. butyricum* in combination with RS should help to further illuminate its potential benefits.

### Conclusion

We have demonstrated that *C. butyricum* is an RS-degrading organism, capable of growing on multiple types and sources of RS, producing substantial amounts of butyrate in the process. It accomplishes this by using an enzyme system that, while being the simplest one found to date in a bacterium capable of degrading RS, exhibits a high degree of synergy and functional diversity in the digestion of RS. The combination of RS digestion and butyrate production in one organism has the potential to bypass the complexities of cross-feeding networks that are normally necessary for the production of butyrate during RS consumption. This unique combination of traits points to its potential utility as part of a symbiotic combination with RS to promote more universal butyrate responses during RS consumption, potentially unlocking some of the health benefits of this prebiotic fiber for a wider population.

## MATERIALS AND METHODS

### Bacterial strain

The bacterial strain used in this study was *C. butyricum* Prazmowski [ATCC 19398, NCTC 7423 (IFO 13949 and VPI 3266)]. It was purchased from the American Type Culture Collection (ATCC) (Manassas, VA, USA), and after establishing growth in Modified Reinforced Clostridial broth, it was maintained in RUM media (see File S1) with 1% (wt/vol) glucose or maltose and 25% (wt/vol) glycerol, stored at −80°C.

### Substrates for growth and enzyme characterization

D-(+)-maltose monohydrate (Sigma-Aldrich) was used to stimulate growth from overnight stocks and as a model substrate for upregulating starch utilization. The maltodextrins used in this study were mixtures of maltodextrins with dextrose equivalent 16.5–19.5 (MD1) (Sigma-Aldrich) and maltodextrins with dextrose equivalent 4.0–7.0 (MD2) (Sigma-Aldrich). The soluble substrates potato starch, pullulan, amylopectin, amylose, dextran, and glycogen were purchased from Sigma-Aldrich (St. Louis, MO, USA), as well as the insoluble cornstarch. The cyclodextrin series (α-, β-, and γ-) were purchased from the American Maize Products Company (Stamford, CT, USA). Native potato starch was purchased from Bob’s Red Mill (Milwaukie, OR, USA). HIGH-MAIZE 260 cornstarch and VERSAFIBE 2470 were provided free of charge by Ingredion, Inc. (Westchester, IL, USA).

### Simulated intestinal digestion of substrates

RS substrates were subjected to simulated intestinal digestion, which served the purpose of emulating the passage of food through the human digestive tract using a modified version of the INFOGEST protocol (61, 62). The protocol was adhered to except for the following modifications. The enzyme addition in the gastric phase was omitted as the small intestinal simulation was most relevant for the substrates tested. Pancreatin from porcine pancreas (Sigma, 8× USP specifications, P7545-500G) was used, and the enzymes were extracted from the powder as follows: briefly, the stock was prepared to 10 mg/mL using 0.1 mM HCl, and the enzymes were extracted for 60 min at 25°C using a VWR tube rotator placed inside a VWR incubating orbital shaker to maintain a constant temperature. Then, centrifugation was performed at 12,000 × *g* for 30 min at 4°C prior to filtering the supernatant through a 0.45-micron syringe filter into a clean 15 mL Falcon tube. The solution was held at 4°C prior to use and was used within 5–7 days of preparation, although activity was found to be retained for up to 5 months under these storage conditions. The volume of extracted pancreatin enzymes was normalized to the α-amylase activity against soluble potato starch of 200 U mL^−1^ targeted in the INFOGEST protocol, as this was most relevant to the purified starches used in this study. The volume of pancreatin extract added to achieve the target α-amylase activity correlated to 7 U mL^−1^ of trypsin activity, with one unit defined as the amount necessary to hydrolyze 1 µmol of *p*-toluene-sulfonyl-l-arginine methyl ester (Acros Organics, Geel, Belgium) per minute at 25°C, pH 8.1. Porcine Pepsin (Sigma-Aldrich) activity was added to reach 2,000 U mL^−1^ based on bovine blood hemoglobin (Sigma-Aldrich) as a substrate, with one unit defined as the amount to produce a change in absorbance at 280 nm of 0.001 per minute at pH 2.0 and 37°C, measured as TCA-soluble products. After the simulated digestion was completed, the RS substrates were prepared by rinsing 2× with sterile water with centrifugation at 4,000 × *g* for 3 min to remove remaining enzymes, then rotated in a VWR Tube Rotator in a 50 mL Falcon tube with 30 mL of 70% ethanol for 16 h. The insoluble cornstarch was prepared similarly except it was not treated with the simulated digestion protocol. Then, the insoluble substrates were washed 10× with 30 mL of sterile water with centrifugation at 4,000 × *g* for 3 min before resuspension in PBS, pH = 6.8 to a final concentration of 2% (wt/vol). The substrates were then tested to ensure no microbial contamination was present by plating 60 µL aliquots in triplicate on RUM media plates (60 × 15 mm) supplemented with 2% (wt/v) maltose and incubated overnight at 37°C.

### Growth experiments

Liquid cultures were grown from frozen stock in RUM media supplemented with 1% (wt/vol) maltose anaerobically in a Coy Anaerobic Chamber at 37°C overnight without shaking. The test samples were then sub-cultured to an optical density (OD) of approximately 0.025 into RUM media supplemented with 1% (wt/vol) of the test substrate (maltose, maltodextrin, corn starch, potato starch, HIGH-MAIZE 260, and VERSAFIBE 2470), with an aliquot removed for downstream analysis of the time zero control point. Cultures were grown anaerobically at 37°C for 8 h (soluble substrates) or 24 h (insoluble substrates) without shaking. Samples were taken at 1, 2, 4, 6, and 8 h for soluble substrates and 6, 10, 12, 20 and 24 h for insoluble substrates.

### Short-chain fatty acid analysis

The samples were obtained by centrifuging 1 mL of spent media from the fermentations at 10,000 × *g* for 1 min and retaining the supernatant, which was frozen at –80°C and thawed on ice prior to analysis. The 250 µL samples were diluted with 10 mM H_2_SO_4_ (Fisher, A510-P212) in a 1:1 ratio and filtered through a 0.45-micron filter into an autosampler vial (the final concentration of H_2_SO_4_ was 5 mM). The ThermoFisher Dionex 5000+ series HPLC was used in the processing of these samples. The system consisted of a 50 mm guard column (Micro-Guard Cation H Cartridge, Bio-Rad, Hercules, CA, USA) followed by a 300 mm ion exclusion column (Aminex HPX-87H, Bio-Rad). Isocratic runs were performed at 50°C with a 4.5 mL/min flow rate, for 60 min. The organic acids were detected by UV absorbance at 214 nm. Standard curves were generated for formate (VWR-BDH Chemicals, BDH4554-500 mL), acetate, propionate, butyrate (Sigma, B103500-100 mL), and lactate (Fisher, A159-500). Co-elution of a RUM media component rendered the quantification of propionate impossible, and lactate was not reported as it was below the limit of quantification in all cases.

### Adherence study

All procedures were performed inside the Coy Anaerobic Chamber. Adherence of *C. butyricum* to cornstarch and the RS granules was determined by methods previously described elsewhere, with slight modifications (15, 63). First, 10 mg of corn starch, agarose, quartz sand, and each of the pre-digested RS substrates were placed into separate sterile 1.5 mL microcentrifuge tubes. These had been cleaned with 70% ethanol and rinsed as per the method described for the cell growth studies. *C. butyricum* was grown overnight in RUM with 1% (wt/vol) maltose and then sub-cultured until exponential growth was achieved and normalized to an OD_600_ of 0.5. One milliliter of this culture was then added to tubes containing each of the starches and the control tube which contained no additional substrate. The cells were given a period of 15 min with agitation at 350 RPM at 37°C using the BioShake IQ to come into contact and adhere to the granules. After 15 min, the control was retained, and the test samples were centrifuged at 700 × *g* for 60 s and the supernatant, representing any non-bound bacterial cells, was removed to a fresh, sterile microcentrifuge tube. Next, the non- and loosely attached bacterial cells were removed by washing with 100 µL each sterile PBS (4×), PBS containing 0.1% Tween 80 (P1754-500 mL) (2×), and then a final wash with PBS. The washes were performed by simply inverting the microcentrifuge tube 3× so as not to physically disrupt adherence to the granules. The centrifugation parameters between each wash were 30 s at 700 × *g*. These washes were added to the non-bound fraction, for a total of 1,700 µL, which was factored into the plate dilutions. Serial dilutions were made using sterile PBS to ensure countable plates were achieved. Strain adherence to the insoluble substrates was determined by quantifying CFUs of the unbound bacteria versus the control on RUM plates supplemented with 1% (wt/vol) maltose after a 16 h incubation period at 37°C. Assays were performed in triplicate and plated in triplicate to account for both technical and biological variations.

### Analysis of starch granules using scanning electron microscopy

The partially fermented insoluble starch granules were isolated from the spent media by centrifugation at 10,000 × *g* for 5 min, washed 3× with sterile, distilled water with centrifugation at 10,000 × *g* for 5 min between each wash, 1× with 1% (vol/vol) bleach for 10 min (only for the fermentation samples), rinsed 2× with distilled water, and 5× with increasing concentrations of ethanol [10%, 20%, 50%, 70%, and 100% (vol/vol)], air-dried, and kept at 4°C until further analysis using SEM. The spent starches from the fermentations and enzyme experiments were retrieved from storage at 4°C and applied to double-sided carbon tapes (Ted Pella, Redding, CA, USA; 168084-1) placed on standard pin stub mounts (Ted Pella; 16111). The carbon tape on the stub was pressed into powder to coat the stub entirely. Loose powder was removed by spraying the sample with canned air. The stub with the sample was placed into a charge reduction sample holder (Phenom World, Waltham, MA, USA), and SEM was then performed on a Phenom G2 Pro instrument (Phenom World, the Netherlands). Images at various locations and magnifications were taken at 5 kilovolts.

### Bioinformatic analysis

#### Enzyme identification and determination of strain homology

To identify the enzyme systems most likely to participate in resistant starch degradation, the CAZy (https://www.cazy.org) database was used to mine the gene annotations from representative *C. butyricum* strains (30). Enzyme systems with a GH13 putative amylase or pullulanase catalytic domain and multiple carbohydrate-binding domains from families shown to interact with starch in other species were selected for further investigation. The genetic material was translated to its protein sequence using the Swiss Institute of Bioinformatics’ Expasy Translate Tool (https://web.expasy.org/translate/), and then SignalP-6.0 (https://dtu.biolib.com/SignalP-6) was used to determine the likelihood of a signal peptide on the N-terminus of the protein sequence, indicating that it is targeted to the extracellular environment (64, 65). The other 17 strains of *C. butyricum* represented in the CAZy database were investigated for the presence of homologous enzymes.

#### Analysis of primary and predicted 3-D structures

Four putative starch-degrading proteins were selected for further analysis, based on the presence of signal sequences. These were designated Amy13A (UniProt: A0A7G5NSF6; GenBank: QMW89799.1), Amy13B (UniProt: A0A7G5NT35; GenBank: QMW90028.1), Pul13A (UniProt: A0A7G5NUT4; GenBank: QMW90627.1), and Amy13C (UniProt: A0A7G5NWM1; GenBank: QMW91264.1), based on their predicted activities. Related GH13 protein sequences were determined using BLASTp’s nr_clustered database (66) (https://blast.ncbi.nlm.nih.gov/Blast.cgi?PAGE=Proteins). The distance trees for results were generated using the Fast Minimum Evolution algorithm, a maximum sequence difference of 0.50 or 0.40 in the case of Pul13A, and the Grishin (protein) evolutionary distance model (67, 68). The AlphaFold (https://alphafold.ebi.ac.uk/) tool was used to generate predicted 3D structures of the enzymes and these were further processed using Pymol (https://pymol.org/2/). Enzyme Amy13B was not available in the AlphaFold database and only the catalytic domain could be successfully predicted using the Phyre2 automatic fold recognition server [(69) http://www.sbg.bio.ic.ac.uk/~phyre2/]. The structure files generated by AlphaFold (70) for enzyme Amy13A were truncated to produce structural and sequence files for each individual CBM for further bioinformatic analyses. These truncated sequences were then subjected to analysis using BLASTp’s nr_clustered database as described above, and distance trees were generated, with the maximum sequence distance set to 0.60. Next, a multiple sequence alignment was performed, against representatives of the various starch-binding CBM families using Clustal Omega (https://www.ebi.ac.uk/Tools/msa/clustalo/), and the resultant tree data were imported to the Interactive Tree of Life (iTol Version 6.7.4) (https://itol.embl.de/) to circularize the rooted phylogenetic tree (71, 72). Similarly, the entire enzyme sequences were aligned with enzymes of other gut-derived starch-degrading bacteria, using iTol to annotate the rooted phylogenetic tree. Furthermore, the Amy13A CBMs were compared to structures in the Protein Data Bank, using the DALI Protein Structure Comparison Server (38).

### Gene cloning

The Expresso T7 Cloning and Protein Expression System (Lucigen, A92701-1) was used. Primers were designed (Table S1) and purchased from Integrated DNA Technologies (IDT, Coralville, IA, USA) to clone the identified enzymes from the *C. butyricum* genome into the pETite N-His Kan vector per the manufacturer’s instructions. The signal sequences were truncated, and a six Histidine tag and TEV protease recognition site were added to the 5′ end. The Phusion Flash High-Fidelity PCR MasterMix (ThermoFisher, F-548L) was used to amplify the identified gene products from the *C. butyricum* genomic material, which had been purified from the other cellular material using the DNeasy PowerLyzer Microbial Kit (Qiagen, Germantown, MD, USA, Cat no. 12255-50). The PCR conditions were as per the Phusion Flash High-Fidelity PCR Master Mix manual guidelines: 10 µL of 2× Phusion Flash High-Fidelity PCR MasterMix, the final concentration of forward and reverse primers was 0.5 µM each, 1 ng of the DNA template, and the volume was made up to 20 µL using Molecular Biology Grade Water (Corning, Manassas, VA, USA, Ref no. 46-000-Cl). PCR was performed using the Eppendorf Mastercycler Gradient thermocycler (Eppendorf, Hamburg, Germany, Model No. 5331) with the following cycling instructions: initial denaturation at 98°C for 10 s followed by 30 cycles of 98°C for 1 s, annealing temperature varied based on the predicted melting temperature (*T*_*M*_) of the cloning primers and was 63.4°C for Amy13A, 61.9°C for Amy13B, 61.2°C for Pul13A, and 63.0°C for Amy13C, and extension was performed at 72°C, and the time varied based on the size of the sequence using 15 s per 1 kb as a guide. A final extension at 72°C was performed for 1 min before the reaction mixture was held at 4°C prior to further usage. Lucigen’s Expresso T7 Cloning and Expression System pETite N-His Kan Vector (Middleton, WI, USA, Part no. A92701-1) was used for ligation-independent cloning into the LGC BioSearch Technologies HI-Control 10G SOLOs chemically competent cells (Hoddesdon, UK, Catalog No. 60110-1) and all steps were completed as per the manufacturer’s instructions. The genetic sequences were confirmed using Sanger Sequencing at the Huck Institute Genomic Core Facility at the Pennsylvania State University.

### Protein expression and purification

#### Protein expression

The plasmids were first transferred into LCG BioSearch Technologies’ HI-Control BL21(DE3) cells; however, Amy13A was the only protein to express well using this cell type. The other three plasmids were then transferred into Agilent’s (Santa Clara, CA, USA) BL21-CodonPlus(DE3)-RIL competent cells per the manufacturer’s instructions. Overproduction of enzyme Amy13A from transformed HI-Control BL21(DE3) cells was performed as follows: 100 mL of Luria-Bertani (LB) broth, Miller (Fisher Scientific) with 30 µg mL^−1^ kanamycin sulfate (Fisher Scientific) was inoculated with the frozen stock and allowed to grow overnight at 37°C with shaking at 225 RPM before sub-culturing at a ratio of 2.5:100 into 3 1/2 liters of fresh LB broth containing 30 µg mL^−1^ kanamycin sulfate and allowed to grow under similar conditions until the mid-log phase was achieved by an optical density reading of ~0.6 at 600 nm, which took ~2.5 h. A sample was removed for downstream analysis and the temperature was decreased to 10°C for 20 min prior to the addition of the induction agent, isopropyl β-D-1-thiogalactopyranoside (Biosynth Carbosynth, Staad, Switzerland), which was added to a concentration of 0.5 mM before shaking at 225 RPM for 16 h at 10°C. An additional sample was removed to analyze via SDS-PAGE and then the cells were pooled and harvested at 10,000 × *g* for 30 min at 4°C prior to storage at −20°C until further use. Overproduction of *C. butyricum* enzymes Amy13B, Amy13C, and Pul13A from transformed BL21-CodonPlus(DE3)-RIL cells was performed similarly, except that both 30 µg mL^−1^ kanamycin and 50 µg mL^−1^ chloramphenicol were added to the growth medium.

#### Protein purification

First, the frozen cell pellets were thawed on ice prior to resuspending in ice-cold IMAC A (25 mM HEPES, 500 mM NaCl, and 20 mM imidazole, pH 8.0) equilibration buffer at a ratio of 10 mL buffer per gram of cells. The cells were lysed using sonication at 20 s intervals, with 30 s of stirring on ice between each burst, for a total of 10 rounds. The solution was then centrifuged at 16,000 × *g* for 30 min at 4°C and then filtered through a 0.45-micron syringe filter. The resulting supernatant was applied to a 1 or 5 mL HisTrap HP (Cytiva, Marlborough, MA, USA) column that had been pre-equilibrated with five column volumes of IMAC A buffer. After loading the protein onto the column, it was washed with IMAC A buffer until a stable baseline was reached (10 column volumes) and then the bound protein was eluted with a linear gradient to 100% IMAC B (25 mM HEPES, 500 mM NaCl, and 250 mM imidazole, pH 8.0). Briefly, for Amy13A, this was achieved using a 1 mL column with a linear gradient across 40 column volumes and 2.5 mL fractions were collected, for Amy13B, Amy13C, and Pul13A, this was achieved using a 5 mL column across 20 column volumes with 2 mL fractions collected. The peak fractions were pooled, and the volume (mL) was recorded. The samples were then concentrated approximately 10-fold using a 30K MWCO centrifugal filtration device. Precipitated protein was removed using centrifugation at 5,000 × *g* for 10 min at 4°C. The proteins were then dialyzed at 4°C against 1 L of reduced-salt IMAC A (25 mM HEPES, 150 mM NaCl, and 20 mM imidazole, pH 8.0) as TEV protease activity is negatively impacted by monovalent salts, with a buffer change after 2 h, then left to dialyze against the same buffer overnight. The His-tagged TEV protease S219V mutant was produced and purified using the pRK793 expression plasmid as previously described, and aliquots were stored at −80°C (73, 74). The *C. butyricum* proteins were then treated with the TEV protease at a ratio of 1 OD_280_ of TEV protease per 100 OD_280_ of recombinant protein for an overnight digest at 4°C, then another 0.25 OD_280_ of TEV protease per 100 OD_280_ was added and allowed to incubate for an additional 6 h at 4°C prior to further purification. The resultant protein solution was then run back through the affinity column and the TEV-cleaved recombinant protein was collected from the flow through and column wash with IMAC A across 15 column volumes. The peak fractions were then pooled, concentrated using the 30K MWCO centrifugal filtration device, and dialyzed against 1 L of Enzyme Buffer (150 mM HEPES, 25 mM NaCl, and 5 mM CaCl_2_ at pH 6.8) overnight at 4°C, with stirring. One buffer change was completed at 2 h, then left to dialyze with the same buffer overnight. The concentration of the protein was estimated spectrophotometrically at 280 nm using the appropriate extinction coefficient.

### Zymogram analysis

*Clostridium butyricum* was inoculated from frozen stock into RUM media containing either 1% glucose or 1% maltose and grown at 37°C in an anaerobic chamber until reaching an OD_600_ of 0.25. The cells were then diluted to an OD_600_ of 0.025 in the same media and grown for 6 h at 37°C in an anaerobic chamber. The cells were then removed by centrifugation (5 min at 10,000 × *g*), and the supernatant was added to 5× SDS-PAGE loading buffer. This was heated for 20 min at 60°C. For comparison, recombinantly produced and purified Amy13A, Amy13B, Amy13C, and Pul13A (see above) were combined and treated similarly. All samples were then loaded onto a 4% polyacrylamide stacking gel (125 mM Tris-HCl pH 6.8, 0.1% SDS) with a 7.5% polyacrylamide/0.2% amylopectin separating gel (375 mM Tris-HCl pH 8.8, 0.1% SDS) and separated for 2.25 h at 100 V at 4°C. The gel was then washed 2× for 25 min at 4°C in 125 mL of 10 mM Tris-HCl pH 7.5, 5 mM 2-mercaptoethanol, 20% 2-propanol, and 5 mM CaCl_2_ and then transferred to 200 mL of 50 mM Tris-HCl pH 6.8, 1 mM EDTA, 2 mM dithiothreitol, and 5 mM CaCl_2_ and incubated at 4°C overnight with light shaking. The gel was then transferred to 200 mL of 50 mM NaPO_4_ pH 6.5 and 5 mM CaCl_2_, incubated for 1 h at 4°C, transferred to a glass plate, wrapped in cling wrap, and incubated at 37°C for 4 h. The gel was then stained with Gram’s iodine, transferred to a lightbox, and photographed.

### Enzyme characterization

The bicinchoninic acid assay was used to determine the release of reducing sugars for all characterization experiments. Briefly, Solution A consisted of 194.2 mg/4.99 mM disodium 2,2′-bicinchoninate, 6 g/0.567 M Na_2_CO_3_ anhydrous, and 2.4 g/0.286 M NaHCO_3_, while Solution B consisted of 124 mg/0.049M CuSO_4_·H_2_O and 126 mg/11.9 M L-serine. These two solutions were stored at 4°C for up to 30 days and were mixed in a 1:1 ratio to make the working solution immediately prior to use. A standard curve was generated using maltose solutions ranging in concentration from 2.5 to 50 µM, and this was used to quantify the reducing sugars generated as compared to a blank reaction that had no enzyme added to it. Samples were diluted prior to adding to the working solution to ensure they were within the linear range of the standard curve. Reactions consisted of 400 µL buffer (25 mM HEPES, 150 mM NaCl, and 5 mM CaCl_2_, pH 7.0), 50 µL 0.3% (wt/vol) substrate, and 50 µL of the enzyme(s). The reactions were performed at 37°C, and the buffer and substrate were allowed to acclimate for 5 min prior to the addition of the enzyme(s). After the incubation period of 15 min for the pH and temperature optimum experiments as well as the characterization on soluble substrates and the 12 h incubation period for insoluble substrates, 50 µL of the reaction mixture was added to 450 µL of the working solution and then heated at 80°C for 30 min to allow the color to develop prior to cooling to room temperature and reading the Abs_560_. Enzyme concentrations used were determined by the amount required to generate 5 U of activity (nmol min^−1^) with their model substrates [Amy13A, Amy13B, and Amy13C = 0.03% (wt/vol) soluble potato starch and for Pul13A = 0.03% pullulan] to be well within the linear range of the assay. Those concentrations were as follows: Amy13A: 3 µg/mL, Amy13B: 0.75 µg/mL, Pul13A: 2.9 µg/mL, and Amy13C: 5.7 µg/mL. Standard curves were generated, and all experiments were performed in triplicate and repeated in two separate instances.

#### pH and temperature optimum experiments

Using the same enzyme concentrations as used for the initial enzyme characterization experiments, new enzyme dilutions were prepared using 150 mM NaCl so as not to influence the pH through the addition of more buffering salts. The universal pH buffers were prepared using stock solutions of 600 mM sodium acetate trihydrate, 600 mM MES, 600 mM HEPES, and 600 mM CHES and filtered through a 0.45-micron filter to remove any undissolved particles that could interfere with absorbance readings. From each buffer, 2.08 mL was added to a 15 mL Falcon tube, and the pH was carefully adjusted to the target so as not to result in solutions of variable ionic strengths (pH 4–pH 10 in 0.5 increments) using 6 M NaOH or HCl, and the final volume was adjusted to 12.5 mL with deionized water. Using the identified pH optimum and appropriate buffer for each enzyme, the temperature range tested was 30°C– 80°C, in 5°C increments. The reactions were incubated at the desired reaction temperature for 5 min prior to the addition of the enzyme.

#### Characterization against soluble substrates

Soluble substrates tested were dextran (negative control), α-, β-, and γ-cyclodextrin, amylose, amylopectin, soluble potato starch, and pullulan. Amylose, amylopectin, and pullulan were first dissolved in 70% DMSO at a 3% concentration before dilution with hot water to a 0.3% final concentration. The other substrates were all directly dissolved in water at 0.3%. All enzyme substrates had 0.02% (wt/vol) NaN_3_ added as a preservative. Each enzyme was tested at the concentration determined from the preliminary experiments against their representative model substrate and all reactions were performed as outlined above for 15 min at 37°C.

#### Characterization against insoluble substrates and synergy study

Substrates were subjected to simulated intestinal digestion as performed in the growth studies but then prepared into suspensions at 0.3% (wt/vol) in the reaction buffer supplemented with 0.02% NaN_3_ as a preservative. Enzyme concentrations used were the same as for soluble substrates. The incubation period was 12 h at 37°C in all cases, as no activity was detected in 15 min and the extended duration was chosen for its biological relevance. After the incubation period, reaction samples were centrifuged at 10,000 × *g* for 30 s to avoid transference of granular material into the working solution that may interfere with the absorbance reading.

#### Analysis of enzyme degradation products

Enzyme assays were set up similarly to the enzyme characterization experiments, except the substrate concentration was increased to 1.5% (wt/vol) to ensure the concentration of degradation products would be sufficient for visualization. Thin layer chromatography was carried out as per Cockburn et al. (75). Briefly, the mobile phase was prepared with 85 mL acetonitrile (Fisher Scientific), 20 mL ethyl acetate, 50 mL 2-propanol (VWR-BDH Chemical), and 60 mL water. The staining solution consisted of 10 mL sulfuric acid added to 190 mL methanol (Fisher Scientific), with 0.6 g N-(1-naphthyl)ethylenediamine dihydrochloride (Sigma-Aldrich) dissolved to produce the final solution.

### Statistical analysis

Minitab 21.2 (64 bit) was used to perform all statistical analyses. Data were tested for normality using the Shapiro-Wilkes test, and non-normal data were subjected to log transformation prior to testing. To compare the means of two groups, a *t*-test was used to investigate the 95% confidence interval for determining significance. When comparing the means of more than two groups, a one-way ANOVA was used to determine if any of the means were different, while a Tukey’s HSD *post hoc* test was used to determine which specific group means, when compared to each other, are different.

## SUPPLEMENTAL MATERIAL

Supplemental figures msphere.00566-23-s0001.docx Fig. S1-S3.

Supplemental methods msphere.00566-23-s0002.xlsx Description of the media used.

Supplemental statistics msphere.00566-23-s0003.xlsx *P*-values for all statistical comparisons in Fig. 7.

Supplemental tables msphere.00566-23-s0004.docx Tables S1-S3.

ASM does not own the copyrights to Supplemental Material that may be linked to, or accessed through, an article. The authors have granted ASM a non-exclusive, world-wide license to publish the Supplemental Material files. Please contact the corresponding author directly for reuse.
