# Supplementary material for: Clostridium butyricum Prazmowski can degrade and utilize resistant starch via a set of synergistically acting enzymes
Source: mSphere. 2023 Dec 22;9(1):e00566-23. doi: 10.1128/msphere.00566-23 (PMC10826348; doi:10.1128/msphere.00566-23)
Supplement: Supplemental figures — Fig. S1-S3. [file msphere.00566-23-s0001.docx]

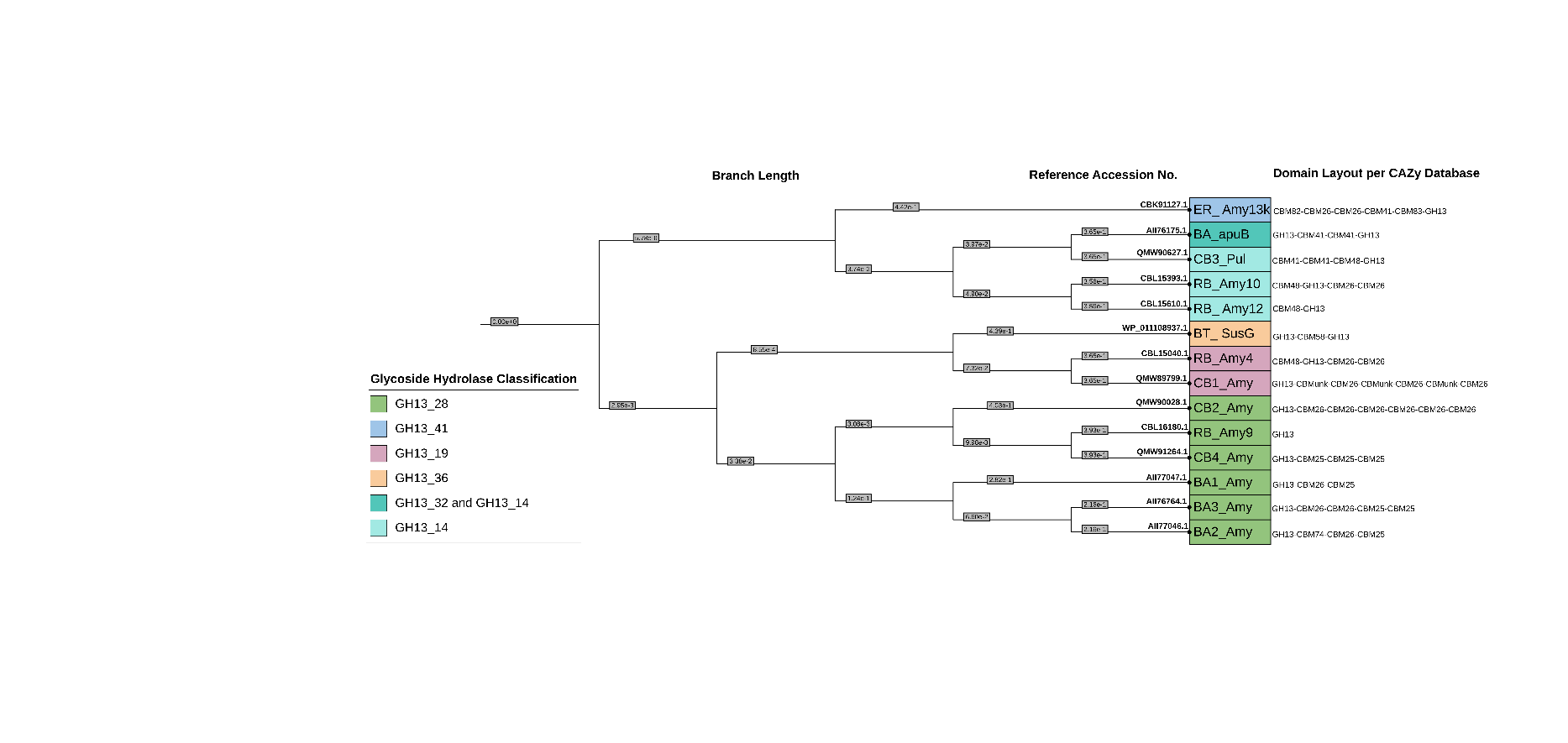


Figure S1. Rooted phylogenetic tree comparing GH13 enzymes from Clostridium butyricum and four other bacterial genomes. C. butyricum Prazmowski (CB) GH13 amino acid sequences (labelled CB1; Amy13A, CB2; Amy13B, CB3; Pul13A, and CB4; Amy13C) are compared with those from other RS degrading species (Ruminococcus bromii L2-63 (RB) and Bifidobacterium adolescentis 22L (BA)) and the non-RS starch degraders (Eubacterium rectale DSM 17629 (ER) and Bacteroides thetaiotaomicron VPI-5482 (BT)). Colors signify the Glycoside Hydrolase Classification as determined using the dbCAN3 Server, or CAZy, in the case of RB_Amy9. The branch lengths, reference accession numbers, and domain layouts as annotated in the CAZy database are displayed above. The tree data was generated using Clustal Omega at EMBL-EBI which was then uploaded to the Interactive Tree of Life (iTOL) to visualize and annotate the figure.


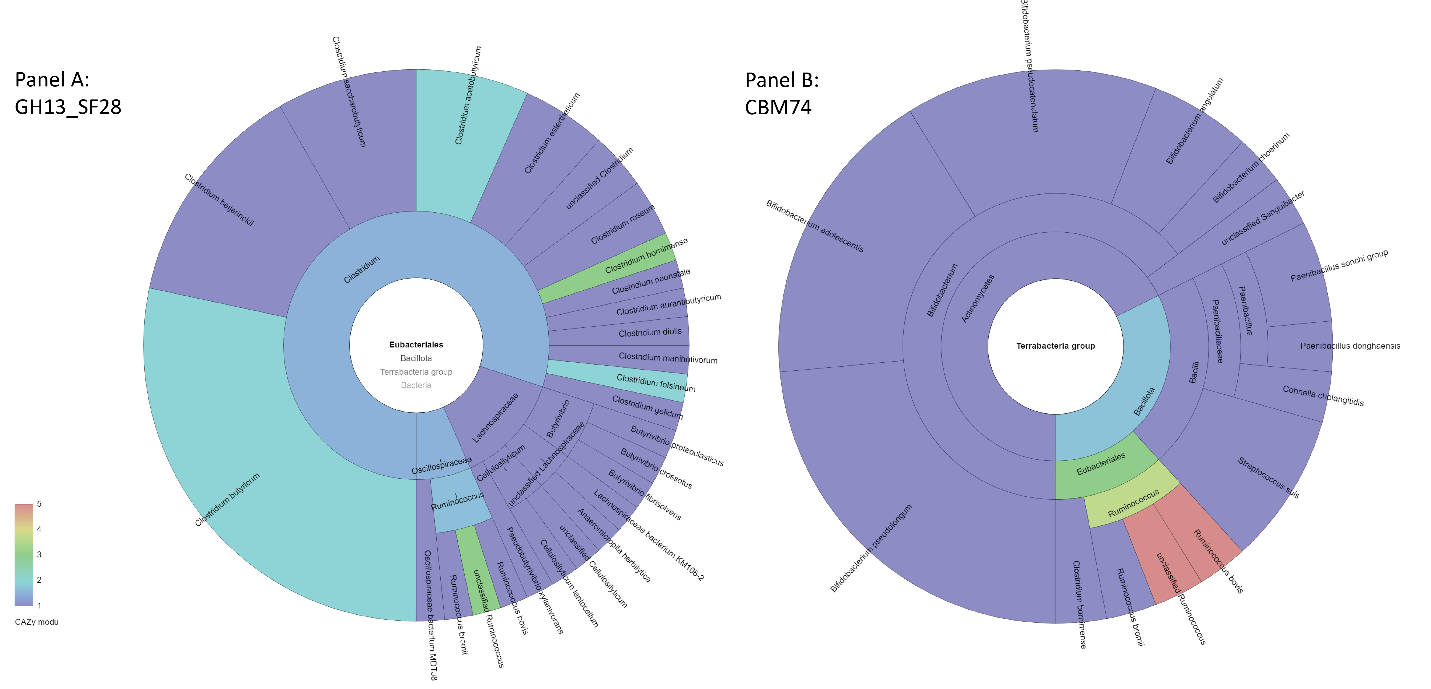


Figure S2. Taxonomic depth of GH13_SF28 and CBM74 distribution. Accessed May 19, 2023 at <http://www.cazy.org/IMG/krona/GH13_28_krona.html> and <http://www.cazy.org/IMG/krona/CBM74_krona.html>.


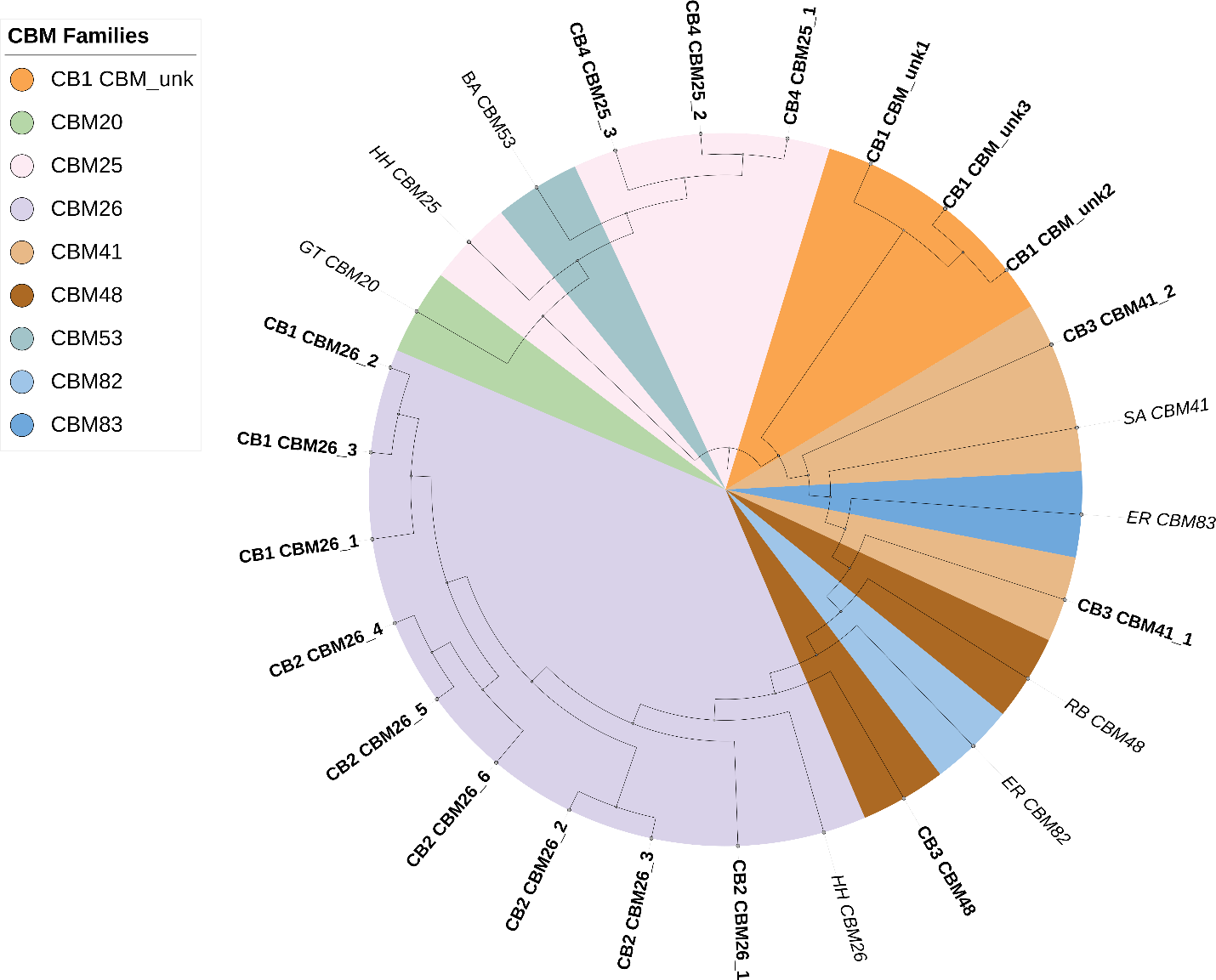


Figure S3. Phylogenetic tree comparing the CBMs from the secreted starch degrading enzymes of Clostridium butyricum Prazmowski (labelled CB1; Amy13A, CB2; Amy13B, CB3; Pul13A, and CB4; Amy13C) and six other bacterial CBMs representative of families known to participate in starch-binding. The C. butyricum sequences are compared other known CBMs either from another RS degrading species: a CBM53 from Bifidobacterium adolescentis 22L (BA), and from the non-RS starch degrader: the CBM82 and CBM83 from Eubacterium rectale DSM 17629 (ER) Amy13k. Included to further put into context the relationship of the unknown CBMs from CB1 are the CBM25 and CBM26 from Halalkalibacterium halodurans C-125 (HH), both of which have a solved crystal structure. The CBM20 from a more distantly related species, Geobacillus stearothermophilus C599 (GT) was also included, as this CBM family has been shown to play a role in non-RS starch binding and this one is part of a maltogenic α-amylase and has a solved structure. The CBM41 from Streptococcus pneumoniae TIGR4 (SA) and CBM48 from Ruminococcus bromii L2-63 (RB) were included, these are each part of a multi-domain pullulanase and have known structures. The tree data was generated using Clustal Omega at EMBL-EBI which was then uploaded to the Interactive Tree of Life (iTOL) to circularize the tree.
