# Supplementary material for: Clostridium butyricum Prazmowski can degrade and utilize resistant starch via a set of synergistically acting enzymes
Source: mSphere. 2023 Dec 22;9(1):e00566-23. doi: 10.1128/msphere.00566-23 (PMC10826348; doi:10.1128/msphere.00566-23)
Supplement: Supplemental tables — Tables S1-S3. [file msphere.00566-23-s0004.docx]

Table S1. Primers used in this study

| **Name** | **Sequence** | **TM** | **Description** |
| --- | --- | --- | --- |
| Amy13A_Fwd | CAT CAT CAC CAC CAT CAC GAG AAC CTG TAC TTC CAG GGC TCT ATT GGG AAA ACT GGT AAC GG | 63.4 | Forward primer for cloning Amy13A into the pETite vector with an N-terminal His-tag and TEV protease site |
| Amy13A_Rev | GTG GCG GCC GCT CTA TTA TTA GTT ATT ATA TTG AGT CCA TGA ACC ATT TTC | 63.7 | Reverse primer for cloning Amy13A into the pETite vector with an N-terminal His-tag and TEV protease site |
| Amy13B_Fwd | CAT CAT CAC CAC CAT CAC GAG AAC CTG TAC TTC CAG GGC GAT ACC ATT CAG GAG AGT CAG G | 62.5 | Forward primer for cloning Amy13B into the pETite vector with an N-terminal His-tag and TEV protease site |
| Amy13B_Rev | GTG GCG GCC GCT CTA TTA CTA TAT CCA TGC ACC ACT TAC ATC | 61.9 | Reverse primer for cloning Amy13B into the pETite vector with an N-terminal His-tag and TEV protease site |
| Pul13A_Fwd | CAT CAT CAC CAC CAT CAC GAG AAC CTG TAC TTC CAG GGC GCA GAG GAA TCT AAT AAG GTA TAC G | 61.5 | Forward primer for cloning Pul13A into the pETite vector with an N-terminal His-tag and TEV protease site |
| Pul13A_Rev | GTG GCG GCC GCT CTA TTA TCA CTT ATT CTT TTC ATA ACT TAT AGT ATC TAC | 61.2 | Reverse primer for cloning Pul13A into the pETite vector with an N-terminal His-tag and TEV protease site |
| Amy13C_Fwd | CAT CAT CAC CAC CAT CAC GAG AAC CTG TAC TTC CAG GGC GCT GAT TCT ACT ACA CTA CCA TCT AAC AC | 65.5 | Forward primer for cloning Amy13C into the pETite vector with an N-terminal His-tag and TEV protease site |
| Amy13C_Rev | GTG GCG GCC GCT CTA TTA TTA TTT AGC TAC AGA ATA ATT GTA ATT AGC TGA ATT ATT AC | 64.6 | Reverse primer for cloning Amy13C into the pETite vector with an N-terminal His-tag and TEV protease site |

Table S2. Protein Homology Across Strains in CAZy Database

| **Strain Designation** | **CBM25s** | **CBM26s** | **CBM41s** | **CBM48s** | **GH13s** | **Reference Accession No.** | **Predicted Domain Layout per CAZy** | **Homologous to CB Enzyme** |
| --- | --- | --- | --- | --- | --- | --- | --- | --- |
| 16-3 | 3 | 9 | 2 | 5 | 20 | QJU43021.1 | GH13-(CBM26)_3_ | Amy13A |
|  |  |  |  |  |  | QJU43232.1 | GH13-(CBM26)_6_ | Amy13B |
|  |  |  |  |  |  | QJU43725.1 | (CBM41)_2_-CBM48-GH13 | Pul13A |
|  |  |  |  |  |  | QJU44491.1 | GH13-(CBM25)_3_ | Amy13C |
| 29-1 | 3 | 9 | 2 | 2 | 15 | QCJ02424.1 | (CBM41)_2_-CBM48-GH13 | Pul13A |
|  |  |  |  |  |  | QCJ02959.1 | GH13-(CBM26)_3_ | Amy13A |
|  |  |  |  |  |  | QCJ03582.1 | GH13-(CBM26)_6_ | Amy13B |
|  |  |  |  |  |  | QCJ03813.1 | GH13-(CBM25)_3_ | Amy13C |
| 1-Apr | 3 | 9 | 2 | 5 | 20 | QCJ05219.1 | (CBM41)_2_-CBM48-GH13 | Pul13A |
|  |  |  |  |  |  | QCJ05706.1 | GH13-(CBM26)_6_ | Amy13B |
|  |  |  |  |  |  | QCJ05919.1 | GH13-(CBM26)_3_ | Amy13A |
|  |  |  |  |  |  | QCJ07723.1 | GH13-(CBM25)_3_ | Amy13C |
| CBUT | 3 | 9 | 2 | 5 | 19 | QUF84740.1 | GH13-(CBM25)_3_ | Amy13C |
|  |  |  |  |  |  | QUF82280.1 | (CBM41)_2_-CBM48-GH13 | Pul13A |
|  |  |  |  |  |  | QUF82755.1 | GH13-(CBM26)_6_ | Amy13B |
|  |  |  |  |  |  | QUF82981.1 | GH13-(CBM26)_3_ | Amy13A |
| CDC_51208 | 3 | 9 | 2 | 5 | 19 | APF23593.1 | GH13-(CBM26)_6_ | Amy13B |
|  |  |  |  |  |  | APF24115.1 | (CBM41)_2_-CBM48-GH13 | Pul13A |
|  |  |  |  |  |  | APF22363.1 | GH13-(CBM25)_3_ | Amy13C |
|  |  |  |  |  |  | APF24486.1 | GH13-(CBM26)_3_ | Amy13A |
| CFSA3987 | 3 | 9 | 2 | 5 | 20 | QGH22310.1 | GH13-(CBM25)_3_ | Amy13C |
|  |  |  |  |  |  | QGH22886.1 | (CBM41)_2_-CBM48-GH13 | Pul13A |
|  |  |  |  |  |  | QGH23398.1 | GH13-(CBM26)_6_ | Amy13B |
|  |  |  |  |  |  | QGH23627.1 | GH13-(CBM26)_3_ | Amy13A |
| CFSA3989 | 3 | 9 | 2 | 5 | 20 | QGH26349.1 | GH13-(CBM25)_3_ | Amy13C |
|  |  |  |  |  |  | QGH26927.1 | (CBM41)_2_-CBM48-GH13 | Pul13A |
|  |  |  |  |  |  | QGH27441.1 | GH13-(CBM26)_6_ | Amy13B |
|  |  |  |  |  |  | QGH27673.1 | GH13-(CBM26)_3_ | Amy13A |
| DS501 | 3 | 9 | 2 | 5 | 18 | QSX04289.1 | GH13-(CBM25)_3_ | Amy13C |
|  |  |  |  |  |  | QSX01769.1 | (CBM41)_2_-CBM48-GH13 | Pul13A |
|  |  |  |  |  |  | QSX02258.1 | GH13-(CBM26)_6_ | Amy13B |
|  |  |  |  |  |  | QSX02470.1 | GH13-(CBM26)_3_ | Amy13A |
| DSM 10702 | 3 | 9 | 2 | 5 | 19 | QMW89799.1 | GH13-(CBM26)_3_ | Amy13A |
|  |  |  |  |  |  | QMW90028.1 | GH13-(CBM26)_6_ | Amy13B |
|  |  |  |  |  |  | QMW90627.1 | (CBM41)_2_-CBM48-GH13 | Pul13A |
|  |  |  |  |  |  | QMW91264.1 | GH13-(CBM25)_3_ | Amy13C |
| JKY6D1 | 3 | 9 | - | 5 | 20 | ALS17193.1 | GH13-(CBM25)_3_ | Amy13C |
|  |  |  |  |  |  | ALS17853.1 | GH13-(CBM26)_6_ | Amy13B |
|  |  |  |  |  |  | ALS18108.1 | GH13-(CBM26)_3_ | Amy13A |
| KNU-L09 | 3 | 9 | - | 5 | 19 | ALP90690.1 | GH13-(CBM25)_3_ | Amy13C |
|  |  |  |  |  |  | ALP91358.1 | GH13-(CBM26)_6_ | Amy13B |
|  |  |  |  |  |  | ALP91612.1 | GH13-(CBM26)_3_ | Amy13A |
| NBRC 13949 | 3 | 9 | 2 | 5 | 19 | BBK76553.1 | GH13-(CBM25)_3_ | Amy13C |
|  |  |  |  |  |  | BBK77255.1 | (CBM41)_2_-CBM48-GH13 | Pul13A |
|  |  |  |  |  |  | BBK77894.1 | GH13-(CBM26)_6_ | Amy13B |
|  |  |  |  |  |  | BBK78132.1 | GH13-(CBM26)_3_ | Amy13A |
| S-45-5 | 3 | 9 | 2 | 5 | 20 | AXB83729.1 | GH13-(CBM26)_3_ | Amy13A |
|  |  |  |  |  |  | AXB83939.1 | GH13-(CBM26)_6_ | Amy13B |
|  |  |  |  |  |  | AXB84426.1 | (CBM41)_2_-CBM48-GH13 | Pul13A |
|  |  |  |  |  |  | AXB85164.1 | GH13-(CBM25)_3_ | Amy13C |
| TK520 | 3 | 9 | - | 5 | 19 | AOR94378.1 | GH13-(CBM25)_3_ | Amy13C |
|  |  |  |  |  |  | AOR94988.1 | GH13-(CBM26)_6_ | Amy13B |
|  |  |  |  |  |  | AOR95182.1 | GH13-(CBM26)_3_ | Amy13A |
| TOA | 3 | 9 | - | 5 | 19 | ANF14313.1 | GH13-(CBM25)_3_ | Amy13C |
|  |  |  |  |  |  | ANF14979.1 | GH13-(CBM26)_6_ | Amy13B |
|  |  |  |  |  |  | ANF15233.1 | GH13-(CBM26)_3_ | Amy13A |

Table 2 shows an analysis of the carbohydrate active enzymes in the genomes of the fifteen strains of *C. butyricum* annotated in the CAZy database as of February 06, 2023. Special attention is called to the number of CBMs in family CBM25, CBM26, CBM41, CBM48, and total GH13 enzymes as compared to the strain in this study, designated DSM 1072 in this table and highlighted in yellow. The strains were analyzed for possible homologs to the enzymes Amy13A, Amy13B, Pul13A, and Amy13C that were cloned and characterized in this study. Enzymes with similar domain layouts are shown above, along with their GenBank reference accession numbers. As seen here, only four of the fifteen strains do not possess the full suite of enzymes, and notably, the missing one is a protein homologous to the Pul13A pullulanase (shaded in grey).

Table S3. Glycoside Hydrolase Family 13 Members of the *Clostridium butyricum* Prazmowski Genome

| **Protein Name** | **Domain Layout** | **GenBank Reference Accession** | **Identifier** | **Definition per NCBI** | **MW (kDa)** |
| --- | --- | --- | --- | --- | --- |
| FF104_00955 (GlgB) | CBM48-GH13 | QMW89567.1 |  | 1,4-alpha-glucan branching protein GlgB | 98684 |
| FF104_00970 | CBM34-GH13 | [QMW89570.1](https://www.ncbi.nlm.nih.gov/entrez/viewer.fcgi?db=protein&val=QMW89570.1) |  | alpha-amylase catalytic domain found in cyclomaltodextrinases and related proteins; cd11338 | 70996 |
| FF104_02235 | GH13-(CBM26)_3_ | [QMW89799.1](https://www.ncbi.nlm.nih.gov/entrez/viewer.fcgi?db=protein&val=QMW89799.1) | Amy13A | starch-binding protein | 128922 |
| FF104_03415 | GH13-(CBM26)_6_ | [QMW90028.1](https://www.ncbi.nlm.nih.gov/entrez/viewer.fcgi?db=protein&val=QMW90028.1) | Amy13B | starch-binding protein | 165700 |
| FF104_03455 (GtfA) | GH13 | [QMW90035.1](https://www.ncbi.nlm.nih.gov/entrez/viewer.fcgi?db=protein&val=QMW90035.1) |  | sucrose phosphorylase | 54708 |
| FF104_06535 (PulA) | (CBM41)_2_-CBM48-GH13 | [QMW90627.1](https://www.ncbi.nlm.nih.gov/entrez/viewer.fcgi?db=protein&val=QMW90627.1) | Pul13A | type I pullulanase | 112869 |
| FF104_06935 (PulA) | GH13 | [QMW90701.1](https://www.ncbi.nlm.nih.gov/entrez/viewer.fcgi?db=protein&val=QMW90701.1) |  | sucrose phosphorylase | 54708 |
| FF104_07780 (GlgX) | CBM48-GH13 | [QMW90863.1](https://www.ncbi.nlm.nih.gov/entrez/viewer.fcgi?db=protein&val=QMW90863.1) |  | glycogen debranching protein GlgX | 80925 |
| FF104_09735 | GH13 | [QMW91230.1](https://www.ncbi.nlm.nih.gov/entrez/viewer.fcgi?db=protein&val=QMW91230.1) |  | alpha-glucosidase | 65549 |
| FF104_09925 | GH13-(CBM25)_3_ | [QMW91264.1](https://www.ncbi.nlm.nih.gov/entrez/viewer.fcgi?db=protein&val=QMW91264.1) | Amy13C | alpha-amylase | 83787 |
| FF104_11075 | GH13 | [QMW91485.1](https://www.ncbi.nlm.nih.gov/entrez/viewer.fcgi?db=protein&val=QMW91485.1) |  | alpha-amylase | 78132 |
| FF104_11565 (GlgB) | CBM48-GH13 | [QMW91580.1](https://www.ncbi.nlm.nih.gov/entrez/viewer.fcgi?db=protein&val=QMW91580.1) |  | 1,4-alpha-glucan branching protein GlgB | 71950 |
| FF104_11845 | CBM34-GH13 | [QMW91634.1](https://www.ncbi.nlm.nih.gov/entrez/viewer.fcgi?db=protein&val=QMW91634.1) |  | alpha-glycosidase | 68042 |
| FF104_13345 | GH13 | [QMW91922.1](https://www.ncbi.nlm.nih.gov/entrez/viewer.fcgi?db=protein&val=QMW91922.1) |  | alpha-glucosidase | 65338 |
| FF104_13515 (PulA) | CBM48-GH13 | [QMW91956.1](https://www.ncbi.nlm.nih.gov/entrez/viewer.fcgi?db=protein&val=QMW91956.1) |  | type I pullulanase | 75445 |
| FF104_13520 | GH13 | [QMW91957.1](https://www.ncbi.nlm.nih.gov/entrez/viewer.fcgi?db=protein&val=QMW91957.1) |  | alpha-amylase | 52663 |
| FF104_16410 | CBM34-GH13 | [QMW92471.1](https://www.ncbi.nlm.nih.gov/entrez/viewer.fcgi?db=protein&val=QMW92471.1) |  | alpha-glycosidase | 68454 |
| FF104_16530 | GH13 | [QMW92495.1](https://www.ncbi.nlm.nih.gov/entrez/viewer.fcgi?db=protein&val=QMW92495.1) |  | alpha-glucosidase | 65625 |
| FF104_19090 (TreC) | GH13 | [QMW93024.1](https://www.ncbi.nlm.nih.gov/entrez/viewer.fcgi?db=protein&val=QMW93024.1) |  | alpha,alpha-phosphotrehalase | 64747 |

As shown above, these are all of the GH13 enzymes in the Clostridium butyricum Prazmowski genome as per the CAZy database. The putative domain layouts and GenBank Accession numbers are provided, along with the predicted biological function and molecular weight (MW) of the proteins. The multidomain starch-binding proteins can have a significantly larger molecular weight than the intracellular processing enzymes. Analysis of these GH13 enzymes using Signal P – 6.0 showed that these are most likely intracellular proteins, except for the four described here as Amy13A, Amy13B, Pul13A, and Amy13C. It is likely the other α-amylase and α-glucosidase enzymes are playing roles in processing the starch degradation products into glucose.
